# Influence of Polymer Hydrophilicity on Solvent-Driven Rare-Earth Element Binding Thermodynamics and Heat Capacity Changes

**DOI:** 10.1021/acs.jpcb.6c02230

**Published:** 2026-10-07

**Authors:** Connor M. B. Gallagher, Rhone B. Jenkins, Robert A. Zalenski, Michael D. Schulz

**Affiliations:** † Department of Chemistry and Macromolecules Innovation Institute, Virginia Tech, Blacksburg, Virginia 24061, United States; ‡ School of Natural and Environmental Sciences, 5994 Newcastle University, Newcastle Upon Tyne NE1 7RU, U.K.

## Abstract

Rare-earth elements (REEs: La–Lu, Y, Sc) are critical to modern technology; however, their acquisition, purification, and separation from each other can be challenging, and current methods are often environmentally damaging. Metal-binding polymers are a promising material class to improve these methods, but fundamental structure–property relationships have yet to be fully developed. Inspired by nature’s use of solvation- and conformation-driven effects to control metal-binding interactions, we hypothesized that modifying the molecular environment around the binding sites on metal-chelating polymers could offer a way to modify solvation structure and direct these interactions. Specifically, we hypothesized that increasing binding-site hydrophilicity would increase the enthalpic favorability of polymer–metal interactions, while maintaining the entropic driving force of metal-ion dehydration. To test this hypothesis, we synthesized a series of poly­(amino acid acrylamides) with systematic changes in the hydrophilicity around the metal-binding sites, using a modular polymer approach to hold key polymer parameters constant. We then used isothermal titration calorimetry (ITC) to measure the solution thermodynamics of polymer–REE binding. Binding by all polymers in this series was entropically driven—consistent with substantial solvent reorganization—but binding site hydrophilicity did not significantly affect binding thermodynamics due to enthalpy–entropy compensation. Interestingly, poly­(glutamic acid acrylamide) displayed a distinct binding behavior, possibly driven by reduced solvent reorganization and larger conformational contributions. In general, lower hydrophilicity was associated with larger desolvation signatures, which could indicate that hydrophobic groups more effectively dehydrate the metal ion than hydrophilic groups. Together, these results demonstrate how polymer structure can modulate desolvation and control polymer-REE binding while also highlighting thermodynamic constraints that limit straightforward tuning of binding affinity, thus providing new insights for the design of materials for critical metal purification.

## Graphical abstract

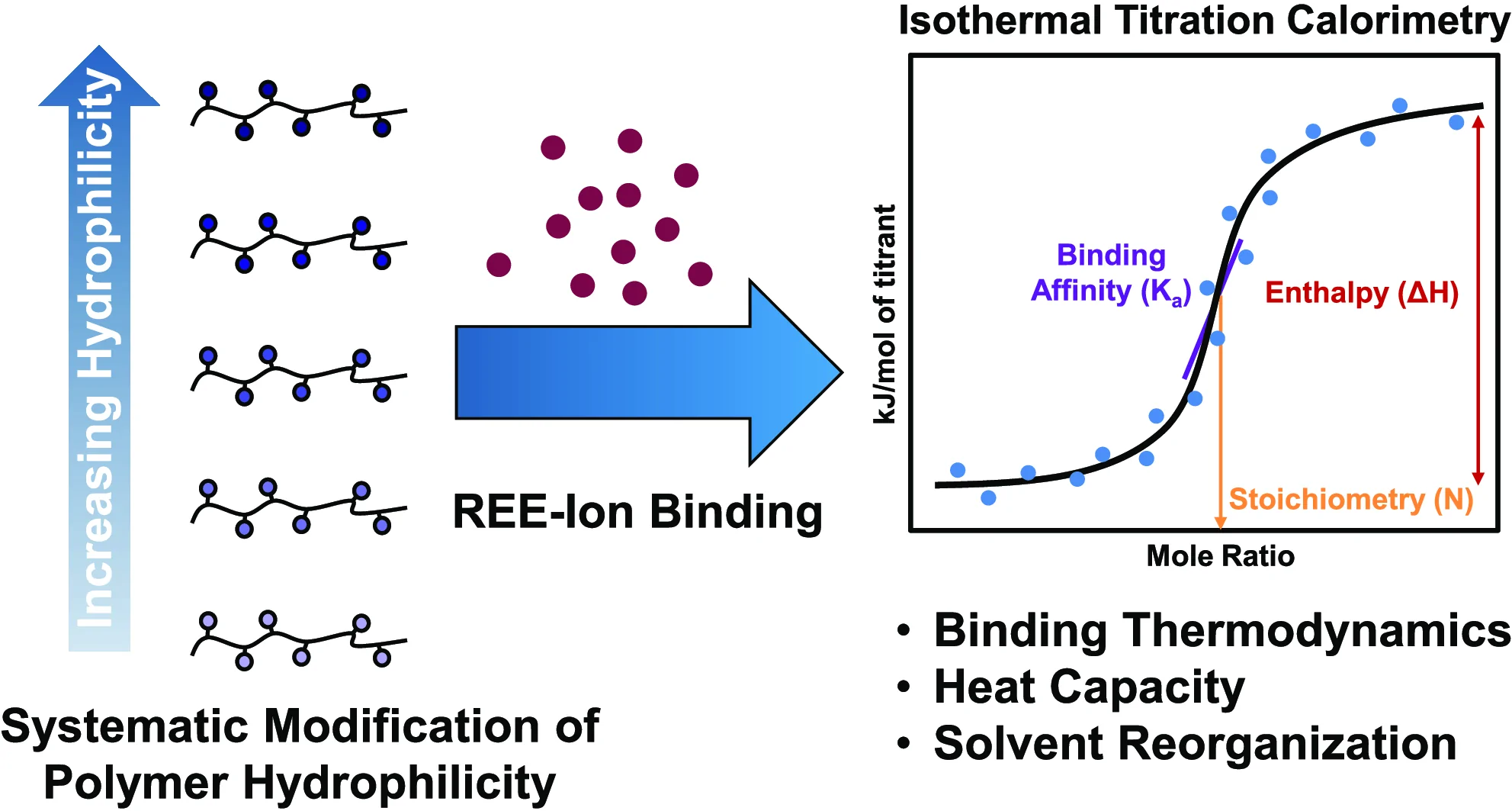

## Introduction

The rare-earth elements (REEs: La–Lu, Y, Sc) are used in a variety of critical energy technologies such as magnets, electric motors, and phosphors.^1–9^ Despite their name, the REEs are not particularly rare, but rather are diffuse throughout the Earth’s crust, making economically viable ore deposits rare and difficult to process.^2,10,11^ Additionally, the REEs exhibit similar chemical properties, further complicating their separation into individual elements.^12,13^ Since the 1960s, these separations have been primarily accomplished via liquid–liquid solvent extraction, but environmental concerns and increasing demand have prompted investigation into other technologies.^14–20^ Due to their tunability, scalability, and low cost, synthetic polymers have emerged as promising materials to improve traditional separations technologies.^21–23^ For example, synthetic polymers are commonly used in the solid phase to extract REEs from a variety of media.^24–30^ As a result of their inherently polyvalent nature, metal-chelating polymers have a higher binding affinity than an analogous small molecule.^31^ This polyvalency has spurred investigation into appending a variety of ligands to polymers; however, structural control has largely been limited to the binding moiety, with comparatively less focus given to the broader polymer structure.

Beyond synthetic polymers, lanthanide-binding proteins, such as Lanmodulin (LanM), display remarkable selectivity for REEs over other metal ions.^32–36^ For example, LanM isolated from *Methylobacterium extorquens* displays a 10^8^-fold preference for REEs over Ca^2+^ despite the latter’s similar ionic radius.^32^ More remarkably, LanM and similar proteins achieve this selectivity using simple carboxylate ligands to interact with the metal ion, rather than the more sophisticated ligands used industrially. Indeed, this selectivity is achieved not by chelating groups themselves, but rather by specific conformational and solvation changes driven by the broader protein structure.^37–41^ Control over desolvation is a common motif across a variety of protein interactions; from metal- and bio­(macro)­molecule-binding to folding and reorganization, nature optimizes desolvation to achieve a variety of functions.^42–47^ However, this same control over (de)­solvation has yet to be fully replicated in synthetic polymer systems, which could potentially combine the selectivity of lanthanide-binding proteins with the low cost and scalability of synthetic polymers.

Inspired by nature’s selectivity, recent work has focused on tuning synthetic polymer structure beyond the chelation site to improve REE separation.^48–51^ For example, Knight and co-workers synthesized a library of copolymers with varying architectures and observed differences in REE binding for compositionally identical polymers with different sequences.^48^ Our group has previously investigated the role of the broader polymer structure in controlling REE-binding using isothermal titration calorimetry (ITC) to directly measure the polymer-metal binding thermodynamics, particularly the entropic contribution driven by solvation and conformational changes.^52–57^ With simple poly­(carboxylate)­s, we found that binding was consistently entropically driven and independent of REE identity.^53^ In particular, we investigated systematically increasing the hydrophobicity of the polymer binding site and found large differences in the thermodynamics of Eu^3+^ chelation, although we observed no clear gain in the overall binding affinity due to enthalpy-entropy compensation (EEC).^54^ Increasing hydrophobicity produced large, positive ΔC_P_ values, consistent with more favorable polar desolvation entropy. These results highlight the power of modulating polymer structure beyond the chelation site to exert thermodynamic leverage on polymer-metal binding interactions.

Building on this work, we recently began studying hydrophilic pendant groups, as they are indispensable for polymeric chelator design. Many of the strongest chelators are multidentate and thus inherently hydrophilic (including multi-dentate carboxylates),^58–61^ and hydrophilic side chains also impart essential solubility and processing benefits, mitigating the aggregation and precipitation that often limit hydrophobic materials in aqueous environments.^62,63^ In nature, metalloproteins and polysaccharides often heavily rely on hydrophilic residues that may provide conformational adaptability and favorable hydrogen-bonding networks.^64–71^ However, previous work, particularly by Sharp and co-workers, has shown that hydrophobic and hydrophilic groups modify hydration structure differently. Hydrophobic groups alter the hydration structure around them by enriching the population of low H-bond angle, “ice-like” waters, while hydrophilic groups instead stabilize a different population of high H-bond angle waters.^72,73^ Displacement of these low-angle, “ice-like” waters tends to produce larger rearrangements of the solute’s first hydration shell and therefore larger entropy changes than displacement of high-angle waters. Using this framework, it may then be expected that including hydrophilic groups around the binding site would reduce the entropic driving force of the reaction. However, the sign of Δ*C*_P_ measured in our previous study indicated that these entropic changes were driven by increasing desolvation of the polar metal ion as hydrophobicity increased, rather than being driven by desolvation of the hydrophobe itself.^54^ Therefore, we hypothesized that hydrophilic groups could still effectively desolvate the metal ion through favorable dipole-ion interactions and thus maintain the interaction’s entropic driving force. These interactions would be more enthalpically favorable and potentially offer a path around enthalpy-entropy compensation while also providing critical data on the role of these indispensable functionalities in polymer-metal binding.

Here, using a modular polymer synthetic approach to control polymer structure and minimize batch-to-batch variations, we investigate how increasing the hydrophilicity of the polymer binding site alters solution-phase REE-polymer binding thermodynamics. Through direct comparison of these results to our prior hydrophobic series, we sought to establish how polarity influences Δ*C*_P_, solvation entropy, and enthalpy–entropy compensation. These insights will provide a more complete picture of how both hydrophobic and hydrophilic pendant groups contribute to polymer–metal interactions and set the stage for designing new chelating polymers that take advantage of hydration sphere differences for critical mineral separations.

## Methods

### Synthetic Methods

Full experimental details are found in the Supporting Information. 2,3,5,6-Tetrafluorophenyl acrylate (TFPA) was synthesized by reaction of 2,3,5,6-tetrafluorophenol with slight excess (1.1 equiv) of acryloyl chloride in DCM and purified by HCl extraction and flash column chromatography. Poly­(TFPA) was synthesized by thermal RAFT polymerization using DDMAT (synthesized as previously reported^54^) as the chain transfer agent. PTFPA was purified by three reprecipitation cycles in THF and chilled methanol. ^1^H and ^19^F NMR and SEC confirmed a well-defined polymer structure. Amino acid acrylamides were synthesized by reaction of PTFPA with an amino acid in DMF in the presence of triethylamine. Samples were purified by dialysis and ^1^H and ^19^F NMR were used to confirm the structure.

### Isothermal Titration Calorimetry

Full experimental details are found in the Supporting Information. ITC experiments were performed on a TA Instrument Affinity-ITC. Stock solutions were prepared by dissolving the polymer or EuCl_3_ in 100 mM acetate buffer and diluted to the desired concentration (Table S1). The polymer solution (350 μL) was placed in the sample cell and the reference cell was filled with DI H2O (300 μL). The first 1.0 μL injection of metal solution was followed by a series of 24, 2.0 μL injections. Each experiment was conducted in duplicate and heats of dilution from control titrations (metal ion into buffer (no polymer present), and buffer into polymer (without metal ion present)) were subtracted before curve fitting. Nonlinear least-squares curve fitting was conducted with the TA Instruments Nano Analyze program and used the TA Instruments Independent model.

## Results and Discussion

### Polymer Synthesis

Modular polymers, i.e., polymers that are easily derivatized using efficient post-polymerization modification reactions, allow for one parent polymer batch to create multiple structurally different daughter polymers while maintaining key polymer parameters such as degree of polymerization, dispersity, and tacticity.^74–77^ A popular approach to modular polymers is the use of activated ester polymers, such as pentafluorophenyl or 2,3,5,6-tetrafluorophenyl acrylates (PFPA or TFPA, respectively), that can undergo quantitative nucleophilic acyl substitution with a variety of nucleophiles.^78–82^ We synthesized TFPA in one step by the reaction of 2,3,5,6-tetrafluorophenol with acryloyl chloride (Figures S1 and S2) and subsequently polymerized it using reversible addition–fragmentation chain transfer (RAFT) polymerization (Scheme 1A). The para-aromatic proton was used to track conversion during polymerization and measure the molecular weight using ^1^H NMR end-group analysis (Figure S4). Size-exclusion chromatography (SEC) and ^1^H and ^19^F NMR spectroscopies confirmed the anticipated polymer structure (Figures S4–S6).

**Scheme 1:**
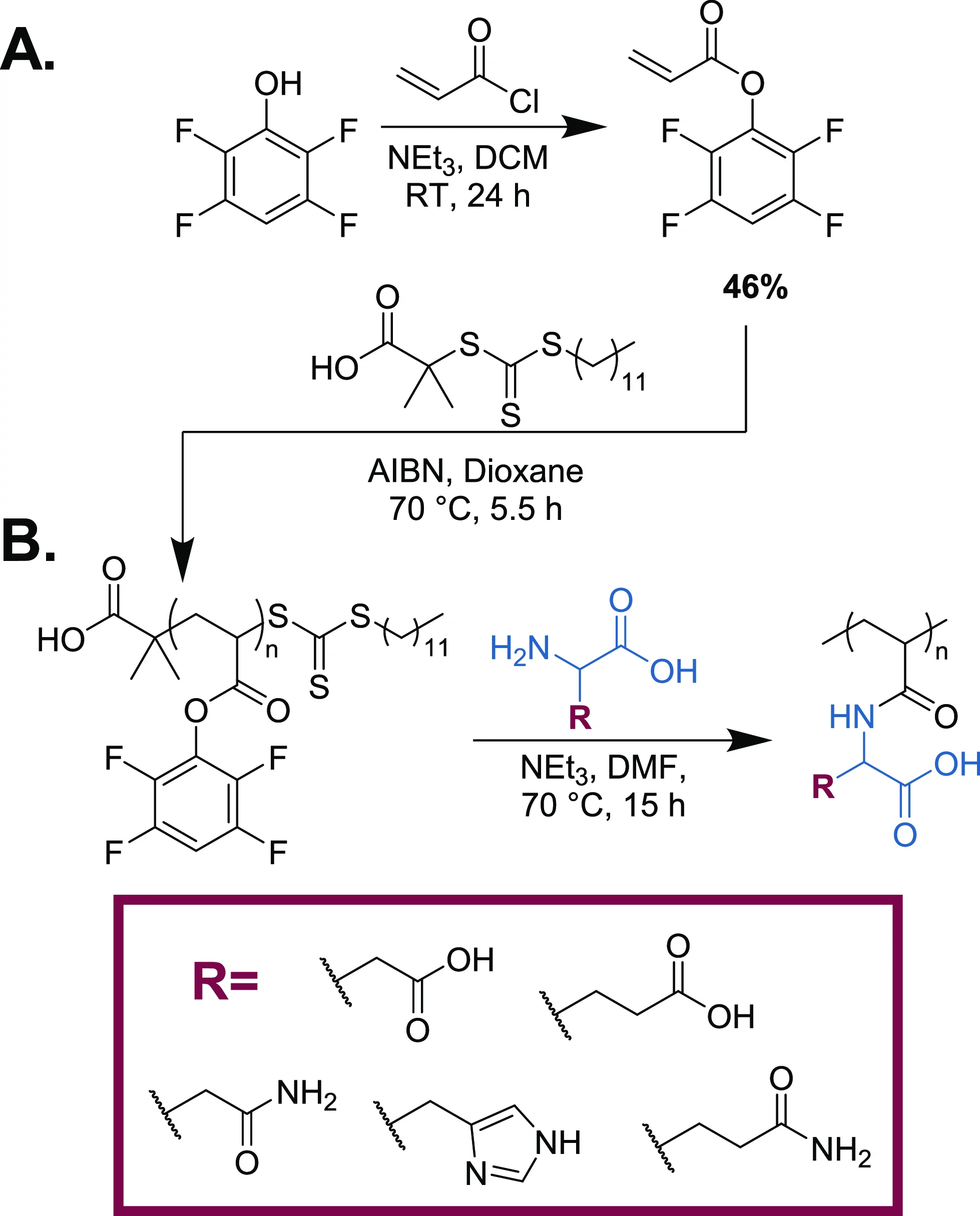
Synthesis and RAFT Polymerization of TFPA (A) and Subsequent Post-Polymerization Modification with Hydrophilic Amino Acids (B)

Poly­(TFPA) was subsequently functionalized using a series of hydrophilic amino acids: aspartic acid, glutamic acid, glutamine, histidine, and asparagine (Scheme 1B). The amine of the amino acids provides a nucleophile to engage in post-polymerization modification while the carboxylic acid can chelate REE ions. The R-groups allow this series of structurally analogous polymers to perturb the molecular environment around the chelation site in a systematic manner. We hypothesized that hydrophilic R-groups would maintain effective metal-ion dehydration—and therefore entropic driving force—without incurring the enthalpic penalty and enthalpy-entropy compensation (EEC) that we previously observed with hydrophobic amino acid acrylamides.^54^ We also attempted to functionalize poly­(TFPA) with lysine and arginine, but the resulting polymers were completely insoluble, perhaps due to crosslinking reactions during the post-polymerization modification or increased aggregation. Following the functionalization reaction, ^19^F NMR spectroscopy confirmed full displacement of the tetrafluorophenyl leaving group before the polymers were purified by dialysis in DI water (Figure S7). Because the amino acid is the only nucleophile in solution, leaving group displacement correlates directly with polymer functionalization. Following dialysis, the hydrophilic poly­(amino acid acrylamides) were largely insoluble in neutral water, likely due to their amphiphilic nature, and so they were characterized in a pH 5, 100 mM acetate buffer. ^1^H and ^19^F NMR spectroscopies confirmed that each post-polymerization modification was successful; we observed characteristic peaks for each amino acid side chain and no signal from the para-aromatic proton, polymer-DMF adduct,^79^ or any ^19^F signals in the final, purified polymer (Figures S8–S12). The five amino acid side chains of this hydrophilic poly­(amino acid acrylamide) series differ only in the structure of the amino acid R-groups, thus enabling systematic comparisons between each different material.

### Binding Thermodynamics

Solution binding thermodynamics between Eu^3+^ and these hydrophilic poly­(amino acid acrylamides) were measured using ITC. Europium (Eu^3+^) was used as a model REE ion in this study as we have previously observed little difference in binding thermodynamics across the REE series using similar polymers.^52,53^ ITC experiments produced thermograms with each peak corresponding to one injection of metal ion (Figures S13–S17). These peaks were then integrated, their areas plotted versus the mole ratio at injection, and the curve fitted to directly calculate the binding thermodynamics (Figure 1).^83–86^ The sigmoidal-shaped binding curves we observed most closely match the so-called “independent model” that describes binding between multiple identical and independent binding sites (eq S1).^87,88^ In some cases, small deviations from the ideal sigmoidal shape were observed at the beginning of the titration, which could be due to interactions between nearby binding sites (e.g., cooperative effects), slight differences between chelation sites on the polymer, or dilution effects, as we have previously observed.^57^ These slight deviations are not uncommon in ITC measurements of polyelectrolytes, and previous results have shown good agreement with the independent model in similar polymers.^53–55,57,89,90^ Curve fitting with the independent model enables the derivation of binding affinity (*K*_a_), binding enthalpy (Δ*H*), and binding stoichiometry. The remaining thermodynamic parameters are calculated according to eq 1, where Δ*G* is the binding free energy and Δ*S* is the binding entropy.

1 $$\Delta G=-\mathrm{RT}\ln\left(K_{a}\right)=\Delta H-T\Delta S$$

**Figure 1:**
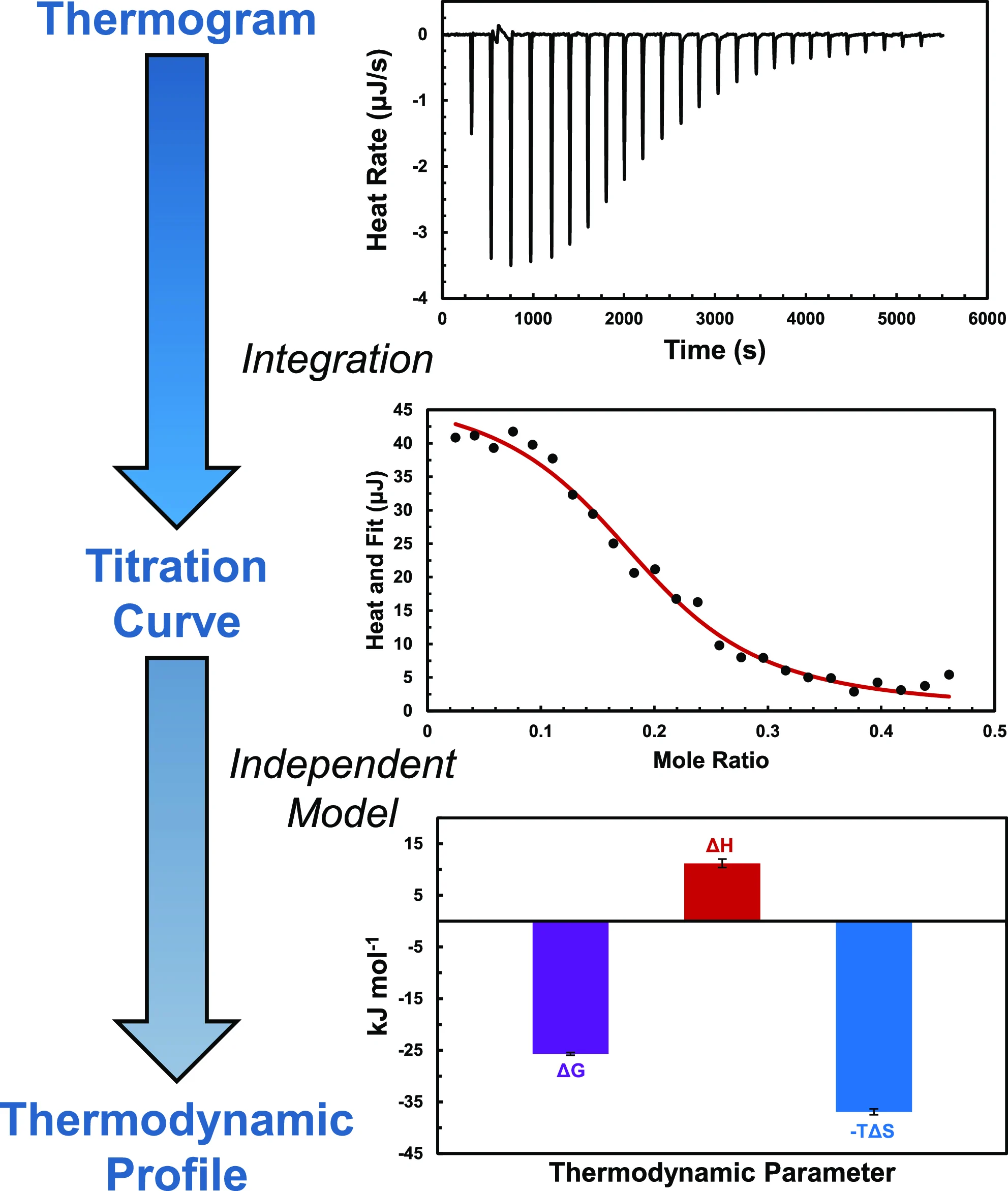
Representative binding thermogram, titration curve, and thermodynamic profile for the interaction between Eu^3+^ and poly­(aspartic acid acrylamide) at 25 °C. Error bars are one standard deviation.

These polymer-Eu^3+^ interactions were all spontaneous (−Δ*G*) while being endothermic (+Δ*H*, enthalpically unfavorable) and driven by a large entropic contribution (+Δ*S*, entropically favorable). This entropically dominated thermodynamic profile is similar to our previous studies of poly­(carboxylates).^52–55,57^ Similar to these previous studies, we hypothesized that this large entropic driving force arises from rearrangement and release of the water molecules that surround the polymer and metal ion, before and after complexation. This profile remained largely consistent as the hydrophilicity of the amino acid R-group was modified. We ordered the polymers by hydrophilicity using the amino acid side chain’s affinity for water,^91,92^ rather than its tendency to be buried within the hydrophobic center of a protein or a distribution coefficient, such as logD or logP (Figure 2). Contrary to our original hypothesis, neither the overall binding affinity (Figure 3A) nor entropic contribution (Figure S18) became noticeably more favorable at higher hydrophilicities. We attributed the consistency in free energy to enthalpy-entropy compensation (EEC), a well-described phenomenon in the protein folding literature, as we observed a linear relationship (*R* ^2^ = 0.893) between enthalpy and entropy for these systems (Figure S19).^93–101^ These data indicate that all hydrophilic groups studied promote comparable metal-ion desolvation, in contrast to our previous work where increasing hydrophobicity corresponded to a more favorable entropy.^54^ This contrast between the hydrophilic and hydrophobic polymer series suggests that although binding is likely driven primarily by metal-ion desolvation, dehydration and solvent rearrangement around the polymer also play a crucial role in the interaction.

**Figure 2:**
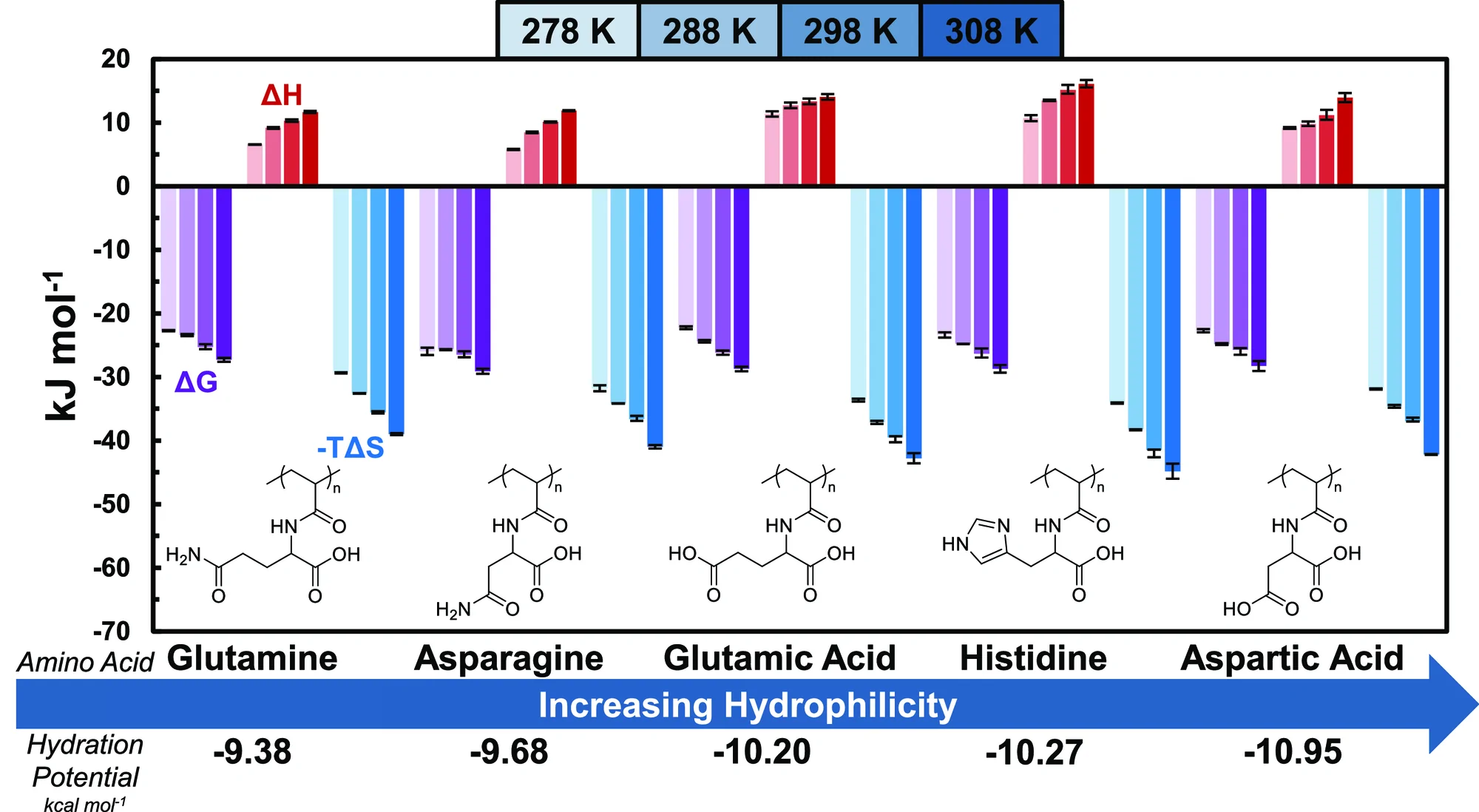
Binding thermodynamics for hydrophilic poly­(amino acid acrylamides) at 5, 15, 25, and 35 °C. Polymers are organized according to increasing magnitude of amino acid side chain hydration potential.^91^ Error bars are one standard deviation.

**Figure 3:**
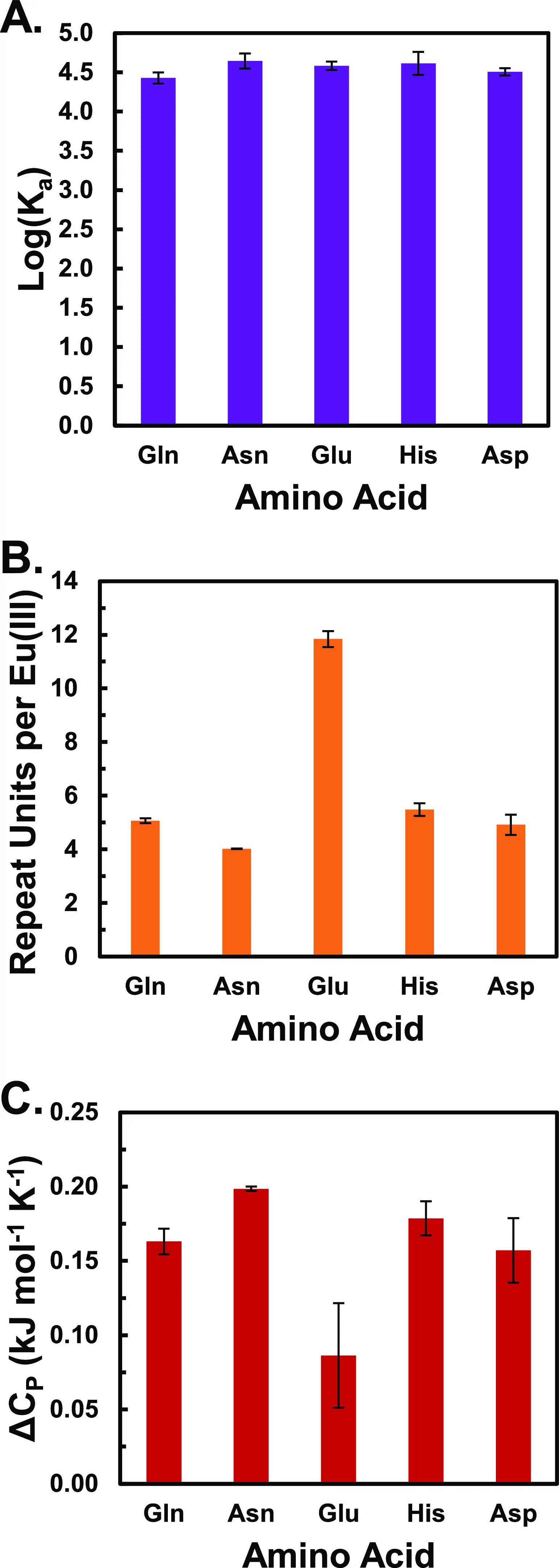
Binding affinity (A) and stoichiometry (B) measured by fitting ITC data for hydrophilic poly­(amino acid acrylamides) binding to Eu^3+^ at 25 °C to the independent model and Δ*C*_P_ values (C) derived from the change in enthalpy as a function of temperature measured by variable temperature ITC. Error bars are one standard deviation.

This observed consistency in the free energy and entropy did not always hold for other binding parameters. The binding stoichiometry was measured directly from fitting experimental data to the independent model and varied noticeably between different poly­(amino acid acrylamides) but did not follow a clear trend with increasing hydrophilicity (Figure 3B). Most poly­(amino acid acrylamides) required between four and six repeat units to bind each europium ion. While larger than what is expected based on charge parity (three carboxylates per Eu^3+^ ion), these values are consistent with previously measured binding stoichiometries for other poly­(carboxylates) and their higher-than-expected values likely reflect inefficiencies in binding due to chain conformation, aggregation, or inaccessible binding sites rather than a crowded binding environment.^52–55,57^ Interestingly, no reduction in the number of repeat units required for binding was observed for poly­(aspartic acid acrylamide) or poly­(glutamic acid acrylamide), despite these polymers containing two chelation-capable carboxylates per repeat unit. We hypothesize that these additional carboxylates could be prevented from aiding in binding due to the polymer conformation or from charge repulsion. Indeed, poly­(glutamic acid acrylamide) required the most repeat units—nearly 12 per Eu^3+^ ion on average. We hypothesized that this increase could be caused by increased steric bulk and flexibility enabling greater charge repulsion, as poly­(glutamic acid acrylamide) also had one of the largest, most unfavorable enthalpic contributions (Figure 2).

### Water Structure

To further explore this consistency in entropy across the series, we investigated the changes to water structure during binding in more detail. Measuring changes in the heat capacity (Δ*C*_P_) during binding has emerged as a powerful tool to study water structure, particularly in the protein folding literature.^72,102–105^ The heat capacity (*C*_P_) is related to all three of the thermodynamic parameters in eq 1 by eq 2.

$$C_{P}={\left(\frac{\partial H}{\partial T}\right)}_{P}=T{\left(\frac{\partial S}{\partial T}\right)}_{P}=-T{\left(\frac{\partial^{2}G}{{\partial T}^{2}}\right)}_{P}$$ 2

Populations of water that are structured differently (e.g., water in a solvation shell versus bulk water) have different heat capacities, and therefore, when the water structure changes (e.g., during binding) there is a change in heat capacity (Δ*C*_P_). Experimentally, we can measure Δ*C*_P_ using variable temperature ITC and eq 3.

3 $${\Delta C}_{P}={\left(\frac{\partial \Delta H}{\partial T}\right)}_{P}$$

Water molecules solvating metal ions and hydrophilic groups have a lower *C*_P_ than bulk water (i.e., water not in a solvation shell), so we hypothesized that increasing the hydrophilicity would increase Δ*C*_P_ as the increasingly hydrophilic groups would have stronger interactions with the metal ion and therefore displace the low *C*_P_ water molecules in the metal’s hydration shell. We performed ITC experiments at four different temperatures (5, 15, 25, and 35 °C; Figure 2) to measure Δ*C*_P_ for our series of hydrophilic poly­(amino acid acrylamides) (Figure 3C). As expected, all materials had a positive ΔC_P_, consistent with desolvation of polar groups during binding as the lower *C*_P_ waters around the hydrophilic side chains, chelating carboxylate, and kosmotropic Eu^3+^ are displaced. Interestingly, no clear trend with regard to hydrophilicity was observed, with both the lowest (poly­(glutamic acid acrylamide), 0.09 kJ mol^–1^ K^–1^) and highest (poly­(asparagine acrylamide), 0.199 kJ mol^–1^ K^–1^) Δ*C*_P_ being observed toward the center of the hydrophilicity series. These Δ*C*_P_ values are generally lower than those previously observed for hydrophobic poly­(amino acid acrylamides).^54^ While the sign of Δ*C*_P_ in both studies indicates that desolvation of polar groups is the primary driving force, the difference in magnitude suggests that dehydration and solvent rearrangement around the polymer also play an important role in determining the binding favorability.

Measurement of the Δ*C*_P_ values also provides insight into the temperature dependence of binding. For an entropically dominated process, one would expect the free energy (and therefore binding affinity) to become more favorable with temperature as higher temperatures weigh the favorable entropic term more (see eq 1). This behavior is observed for all the hydrophilic poly­(amino acid acrylamides) except for poly­(asparagine acrylamide) (Figure S20). Poly­(asparagine acrylamide) has the highest Δ*C*_P_, meaning that the unfavorable enthalpic term increases the most with increasing temperature (see eq 3), nearly canceling out the entropically driven increase in binding affinity in this case. Consequently, modifying the polymer structure around the binding site can change an important physical behavior (the temperature dependence of binding affinity) without modifying the chelating group itself.

To further investigate the changes to the entropic contribution, we turned to a method first described in the protein-folding literature to deconvolute the contributions to the entropy arising from different sources.^42,106–111^ Using this approach, we assumed the experimental entropy (Δ*S*_Exp_) to be composed of three main contributions: the rotational-translational (Δ*S*_RT_), conformational (Δ*S*_Conf_), and solvation (Δ*S*_Solv_) entropies (eq 4).

4 $${\Delta S}_{\mathrm{Exp}}={\Delta S}_{RT}+{\Delta S}_{\mathrm{Co}nf}+{\Delta S}_{Solv}$$

5 $${\Delta S}_{RT}=R\,\;\ln\left(\frac{1}{\left[H_{2}O\right]}\right)$$

6 $${\Delta S}_{Solv}=\Delta C_{P}\,\;\ln\left(\frac{T_{\exp}}{T_{Ref}}\right)$$

We then assumed the Δ*S*_RT_ to be solely a function of the concentration of the solvent (55.6 M for water) and therefore to be constant for a given solvent (eq 5). Next, the Δ*S*_Solv_ can be calculated using eq 6 from the experimental temperature (*T*_Exp_), the Δ*C*_P_ value, and an empirically derived temperature (*T*_Ref_) where solvation entropy extrapolates to zero.^42,107^ Finally, since the Δ*S*_Exp_ is determined by the ITC experiment, the Δ*S*_Conf_ can be calculated by difference using eq 4.

Two literature reference temperatures (eq 6) can be plausibly considered for entropy decomposition in these polymer–metal systems: a reference near 176 K associated with polar hydration entropy, which is chemically intuitive for hydrophilic polymers and metal-desolvation-dominated interactions,^107,112–114^ and a temperature near 386 K associated with hydrophobic (de)­solvation, originally derived by studying the burial of hydrophobic surface during protein folding.^42,107,111^ Because the measured entropy of polymer–lanthanide binding likely reflects coupled contributions from metal-ion desolvation, polymer-side-chain hydration, and polymer conformational reorganization, neither value should be regarded as uniquely valid for this system. Instead, calculations based on 176 or 386 K are best interpreted as alternative model assumptions that reflect distinct mechanistic possibilities rather than as definitive measures of solvation entropy. Here, we use 176 K as *T*_Ref_ because the uniformly positive ΔC_P_ values and similar thermodynamic profiles across the polymer series suggest that binding is broadly governed by solvent reorganization around polar groups. However, this model necessarily neglects contributions from non-polar solvation changes (e.g., polymer backbone interactions) and assumes that all solvation changes occur around polar surfaces.

The relationships in eqs 4–6 enable examination of the relative contributions of different sources of entropy to the system (Figure 4); however, this approach does not uniquely determine the independent physical contributions to the total entropy. Because this decomposition relies on an assumed reference temperature and simplified treatments of heat capacity contributions, the resulting partitioned entropy terms are inherently model-dependent and should be interpreted cautiously. Accordingly, we use this analysis as a qualitative framework to guide mechanistic interpretation, rather than as a quantitative determination of distinct entropy components.

**Figure 4:**
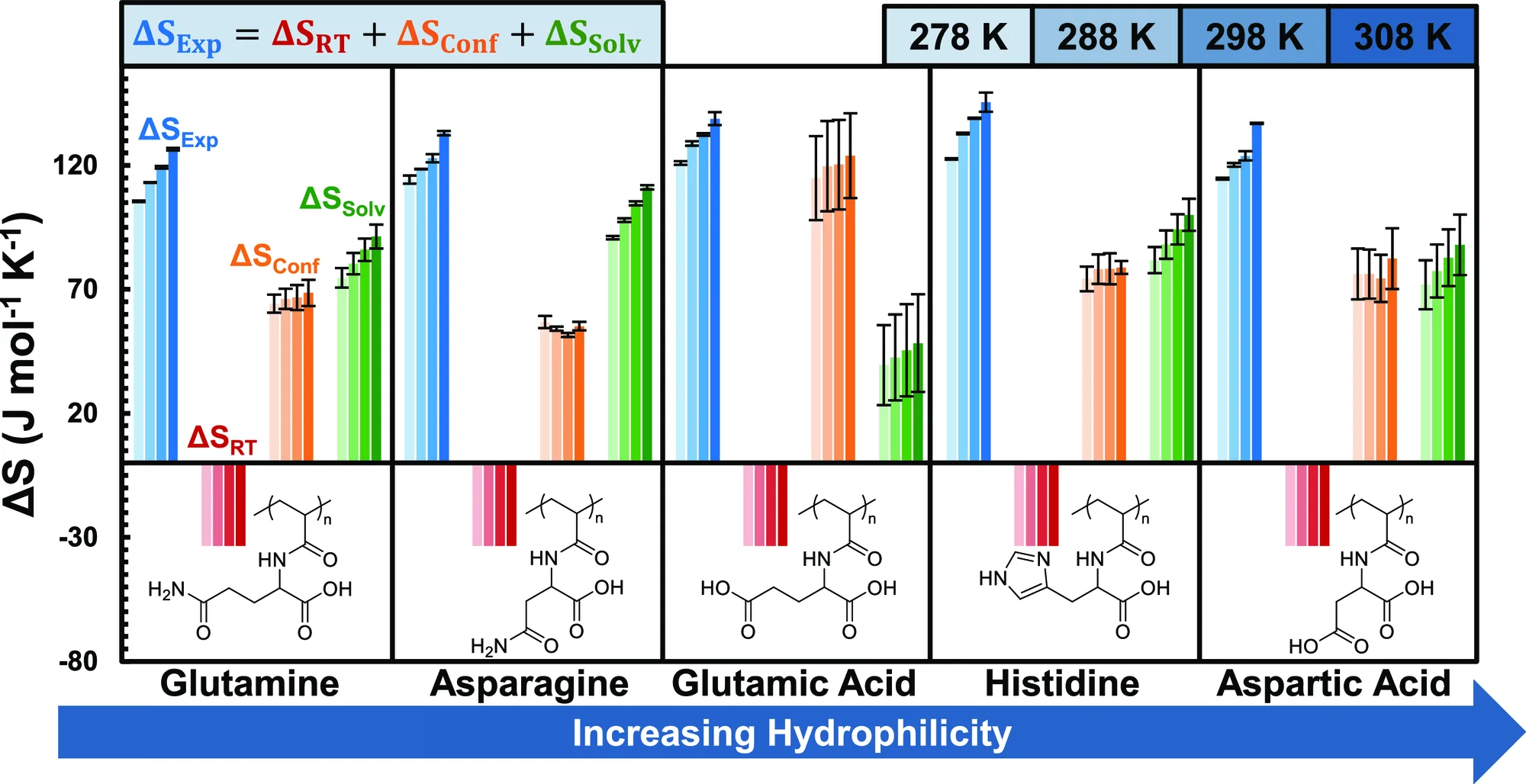
Entropic contributions to binding for hydrophilic poly­(amino acid acrylamides) interaction with Eu^3+^ at 5, 15, 25, and 35 °C. Calculations are based on eqs 4–6 and assume a reference temperature of 176 K. See main text for a discussion of the assumptions underpinning these values. Error bars are one standard deviation.

Under this model, only rotational–translational entropy was unfavorable for all hydrophilic poly­(amino acid acrylamides), while both conformational and solvation entropy contributed favorably. Additionally, the overall increase in experimentally measured entropy with temperature was driven largely by an increase in solvation entropy with temperature (see eq 6), as the other contributions did not vary significantly with temperature. Both poly­(glutamic acid acrylamide) and poly­(aspartic acid acrylamide) had higher apparent conformational entropies than the corresponding amides, possibly indicating more favorable binding conformations due to the second carboxylate. However, because there was little improvement in binding affinity or stoichiometry moving from the amide to the acid (Figure 3A,B), we hypothesize that this apparent gain in conformational entropy could be due to more favorable interpolymer hydrogen bonding interactions, rather than increased polyvalent metal binding. Running these calculations with the alternative 386 K reference temperature produces results that partition the entropy into a negative solvation contribution offset by a dominant conformational term. Such a partitioning runs counter to chemical intuition and reinforces our decision to use 176 K as the reference temperature for this analysis.

Interestingly, poly­(glutamic acid acrylamide) had a notably lower Δ*C*_P_ (and therefore a lower apparent solvation entropy), indicating that this polymer–metal interaction included relatively few changes to solvation structure compared to the other polymers. It was also the only case where apparent conformational entropy contributed more than solvation entropy. When combined with the highly inefficient stoichiometries measured, this finding indicates that while the binding conformations adopted are very favorable (high conformational entropy), they involve few carboxylate groups per polymer interacting with the Eu^3+^ ion (high stoichiometry). We hypothesize that this outlying behavior could be due to a change in driving force, from maximizing desolvation to minimizing conformational penalties via minimizing charge–charge repulsion and steric hindrance.

Contrary to our original hypothesis, desolvation entropy did not appear to increase with side chain hydrophilicity and indeed the largest solvation entropies and Δ*C*_P_ values were observed for one of the more hydrophobic side chains (Asn acrylamide). Under the entropy deconvolution model we employed, at lower hydrophilicities (Gln and Asn acrylamides) the conformational entropy contributes less than the solvation entropy, while at higher hydrophilicities (Glu and Asp acrylamides) the conformational entropy contributes equally or more than solvation entropy. We hypothesized that the higher desolvation entropy and larger Δ*C*_P_ values at lower hydrophilicities could be due to the more hydrophobic groups desolvating the metal ions to a greater degree. Our previous study^54^ of hydrophobic amino acid acrylamides also showed an increase in Δ*C*_P_ (and therefore solvation entropy, see eq 6) with lower hydrophilicities, and a study of amphiphilic copolymers reported increased desolvation with higher hydrophobic content.^48^ Taken together, both these results suggest that hydrophobic groups around the binding site promote larger solvation changes upon binding by more effectively dehydrating the metal ion. The interactions with the metal ion of the hydrophilic groups studied in this work appear to produce smaller solvation changes and therefore exert less thermodynamic leverage over the interaction.

## Conclusions

In conclusion, we synthesized a series of hydrophilic poly­(amino acid acrylamides) to systematically vary the polymer structure and hydration environment around the chelation site. We studied how these structural changes influence polymer-REE binding thermodynamics and observed similar, entropically driven thermodynamic profiles across the series, independent of amino acid hydrophilicity, likely due to enthalpy–entropy compensation. Positive Δ*C*_P_ values are consistent with these interactions being driven by polar desolvation, while slight differences in the Δ*C*_P_ across the series suggest shifts in the relative contributions of apparent (de)­solvation and conformational entropy with changing hydrophilicity. Notably, poly­(glutamic acid acrylamide) exhibited distinct behavior, consistent with greater changes in conformational entropy than the other polymers, possibly indicating an alternate driving force than maximizing desolvation that merits further investigation. Overall, these results demonstrate that the broader polymer structure plays an important role in governing solvent reorganization and binding thermodynamics and provide new insights into hydration-driven routes for the design of polymeric materials for critical metal separation and extraction.

## Supplementary Material



## Notes

Full experimental procedures, NMR spectra, ITC thermograms, and additional figures (PDF)
