# Supplementary material for: Influence of Polymer Hydrophilicity on Solvent-Driven Rare-Earth Element Binding Thermodynamics and Heat Capacity Changes
Source: J Phys Chem B. 2026 Oct 7;130(42):11286–95. doi: 10.1021/acs.jpcb.6c02230 (PMC13699996; doi:10.1021/acs.jpcb.6c02230)
Supplement: Supplementary file 1 [file jp6c02230_si_001.pdf]

Supporting Information for "Influence of Polymer Hydrophilicity on Solvent-Driven Rare-Earth Element Binding Thermodynamics and Heat Capacity Changes"

Connor M.B. Gallagher,[a,b] *Rhone B. Jenkins,*[a] *Robert A. Zalenski II,*[a] *and Michael D. Schulz*[a]*

a: Department of Chemistry and Macromolecules Innovation Institute, Virginia Tech, Blacksburg, VA, USA 24061

b: Current affiliation: School of Natural and Environmental Sciences, Newcastle University, Newcastle Upon Tyne NE1 7RU, UK

Materials and Characterization S3

Synthetic Methods S4

Synthesis of 2,3,5,6-tetrafluorophenyl acrylate (TFPA): S4

Figure S1. ^{1}H NMR (400 MHz) spectrum of tetrafluorophenyl acrylate (TFPA) in $CDCl_3$. S5

Figure S2. ^{19}F NMR (375 MHz) spectrum of tetrafluorophenyl acrylate (TFPA) in $CDCl_3$ S5

Synthesis of 2-(Dodecylthiocarbonothioylthio)-2-methylpropionic acid (DDMAT): S6

Figure S3. ^{1}H NMR (400 MHz) spectrum of DDMAT in $CDCl_3$. S7

RAFT Polymerization of TFPA: S8

Figure S4. ^{1}H NMR (400 MHz) spectrum of poly(tetrafluorophenyl acrylate) (PTFPA) in $CDCl_3$. S9

Figure S5. ^{19}F NMR (375 MHz) spectrum of poly(tetrafluorophenyl acrylate) (PTFPA) in $CDCl_3$. S10

Figure S6. SEC light scattering trace of poly(tetrafluorophenyl acrylate) (PTFPA) in DMAc with 50 mM LiCl. S10

Representative Post-Polymerization Modification (poly(aspartic acid acrylamide)): S11

Figure S7. ^{19}F NMR (375 MHz, DMSO-d_6) spectra of PTFPA before (A) and after functionalization by glutamine (B), asparagine (C), glutamic acid (D), histidine (E), and aspartic acid (F) showing full displacement of the tetraflourophenyl leaving group. S11

Figure S8. ^{1}H (600 MHz, D_2O, above) and ^{19}F NMR (375 MHz, D_2O, below) spectra of poly(glutamine acrylamide) in pH 5, 100 mM acetate buffer. S12

Figure S9. ^{1}H (600 MHz, D_2O, above) and ^{19}F (375 MHz, D_2O, below) NMR spectra of poly(asparagine acrylamide) in pH 5, 100 mM acetate buffer. S13

Figure S10. ^{1}H (600 MHz, D_2O, above) and ^{19}F (375 MHz, D_2O, below) NMR spectra of poly(glutamic acid acrylamide) in pH 5, 100 mM acetate buffer. S14

Figure S11. ^1H (600 MHz, D_2O, above) and ^{19}F (375 MHz, D_2O, below) NMR spectra of poly(histidine acrylamide) in pH 5, 100 mM acetate buffer. S15

Figure S12. ^1H (600 MHz, D_2O, above) and ^{19}F NMR (375 MHz, D_2O, below) spectra of poly(aspartic acid acrylamide) in pH 5, 100 mM acetate buffer. S16

Isothermal Titration Calorimetry S17

Table S1. Optimized ITC concentrations. Polymer concentrations are given as repeat unit concentrations. S18

Table S2. Thermodynamics of Eu^{3+} binding by hydrophilic poly(amino acid acrylamides). S19

Figure S13. ITC thermograms for Eu^{3+} binding by poly(glutamine acrylamide) at 5(A & B), 15(C & D), 25(E & F), and 35(G & H) °C. S20

Figure S14. ITC thermograms for Eu^{3+} binding by poly(asparagine acrylamide) at 5(A & B), 15(C & D), 25(E & F), and 35(G & H) °C. S21

Figure S15. ITC thermograms for Eu^{3+} binding by poly(glutamic acid acrylamide) at 5(A & B), 15(C & D), 25(E & F), and 35(G & H) °C. S22

Figure S16. ITC thermograms for Eu^{3+} binding by poly(histidine acrylamide) at 5(A & B), 15(C & D), 25(E & F), and 35(G & H) °C. S23

Figure S17. ITC thermograms for Eu^{3+} binding by poly(aspartic acid acrylamide) at 5(A & B), 15(C & D), 25(E & F), and 35(G & H) °C. S24

Figure S18. ΔG (purple circles) and -TΔS (blue diamonds) for Eu^{3+} binding by hydrophilic poly(amino acid acrylamides) as a function of amino acid side chain hydration potential.[5] Error bars are one standard deviation. S25

Figure S19. -TΔS vs ΔH for Eu^{3+} binding by hydrophilic poly(amino acid acrylamides). Error bars are one standard deviation. S25

Figure S20. Log (K_a) at 5, 15, 25, and 35 °C for Eu^{3+} binding by hydrophilic poly(amino acid acrylamides). Error bars are one standard deviation. S26

References: S27

Materials and Characterization

Triethylamine, glutamine, asparagine, glutamic acid, histidine, aspartic acid, tribasic potassium phosphate (K_3PO_4), and carbon disulfide (CS_2) were purchased from Oakwood Chemical. Dichloromethane (DCM), 12.1N HCl, hexanes, acetone, tetrahydrofuran (THF), methanol (MeOH), and N,N-dimethylformamide (DMF) were purchased from Fisher Scientific. Dodecanethiol, 2-bromo-2-methylpropionic acid, azobisisobutyronitrile (AIBN), 1,4-dioxane, and 1,3,5-trioxane were purchased from Sigma Aldrich. 2,3,5,6-Tetrafluorophenol was purchased from Synquest. Acryloyl chloride was purchased from Alfa Aesar. Deuterated solvents were purchased from Cambridge Isotope Labs. SnakeSkin™ dialysis tubing (16mm I.D., 3.5 kDa MWCO) was purchased from ThermoFisher. AIBN was recrystallized from MeOH prior to use. All other chemicals were used as received.

^{1}H NMR were acquired on an Agilent MR4 400 MHz or Neo 600 MHz (equipped with Prodigy Cryoprobe) spectrometer. Chemical shifts in the ^{1}H NMR were referenced to the residual solvent resonance signals. ^{19}F NMR was acquired on a 400 MHz JEOL system.

Size exclusion chromatography (SEC-MALS) was performed using a mobile phase consisting of N,N-dimethylacetamide (DMAc) with 50 mM LiCl at a rate of 1.0 mL/min at 40 °C on two Agilent Technologies PLgel 10 µm MIXED-B LS 300 x 7.5 mm columns connected in series with Wyatt Technologies miniDAWN TREOS II light scattering and Optilab T-REX differential refractive index (dRI) detectors. Data analysis was performed using Astra version 7.2.2.10 software (Wyatt Technologies). The dn/dc value of poly(tetrafluorophenylacrylate) was measured as 0.0477 mL/g.

Synthetic Methods

Synthesis of 2,3,5,6-tetrafluorophenyl acrylate (TFPA):

2,3,5,6-Tetrafluorophenol (83.74g, 504 mmol, 1.0 equiv.) was placed in a 500 mL 3-neck round bottom flask that was charged with a magnetic stir bar and attached to an addition funnel. The tetrafluorophenol was then dissolved in approximately 400 mL of DCM and placed under a N_2 atmosphere. Triethylamine (106 mL, 756 mmol, 1.5 equiv.) was added by syringe in one portion and the solution was stirred in an ice bath for 20 minutes. Acryloyl chloride (44.83 mL, 554.4 mmol, 1.1 equiv.) was added dropwise through the addition funnel over the course of approximately 30 minutes and the reaction was stirred at room temperature overnight. The crude reaction was filtered into a separatory funnel and washed with three, 200 mL portions of 3M HCl and three, 200 mL portions of saturated sodium chloride solution. The organic layer was dried over $MgSO_4$, filtered, and the solvent was removed by a rotary evaporator. The crude product was then purified by automated flash column chromatography using silica gel and a hexanes/acetone gradient and dried *in vacuo* to isolate the product as a colorless oil (51.0g, 46% yield).

^{1}H NMR (400 MHz, $CDCl_3$, δ): 7.0 (m, 1H), 6.7 (dd, 1H), 6.4 (dd, 1H), 6.1 (dd, 1H)

^{19}F NMR (375 MHz, $CDCl_3$, δ): -139, -153

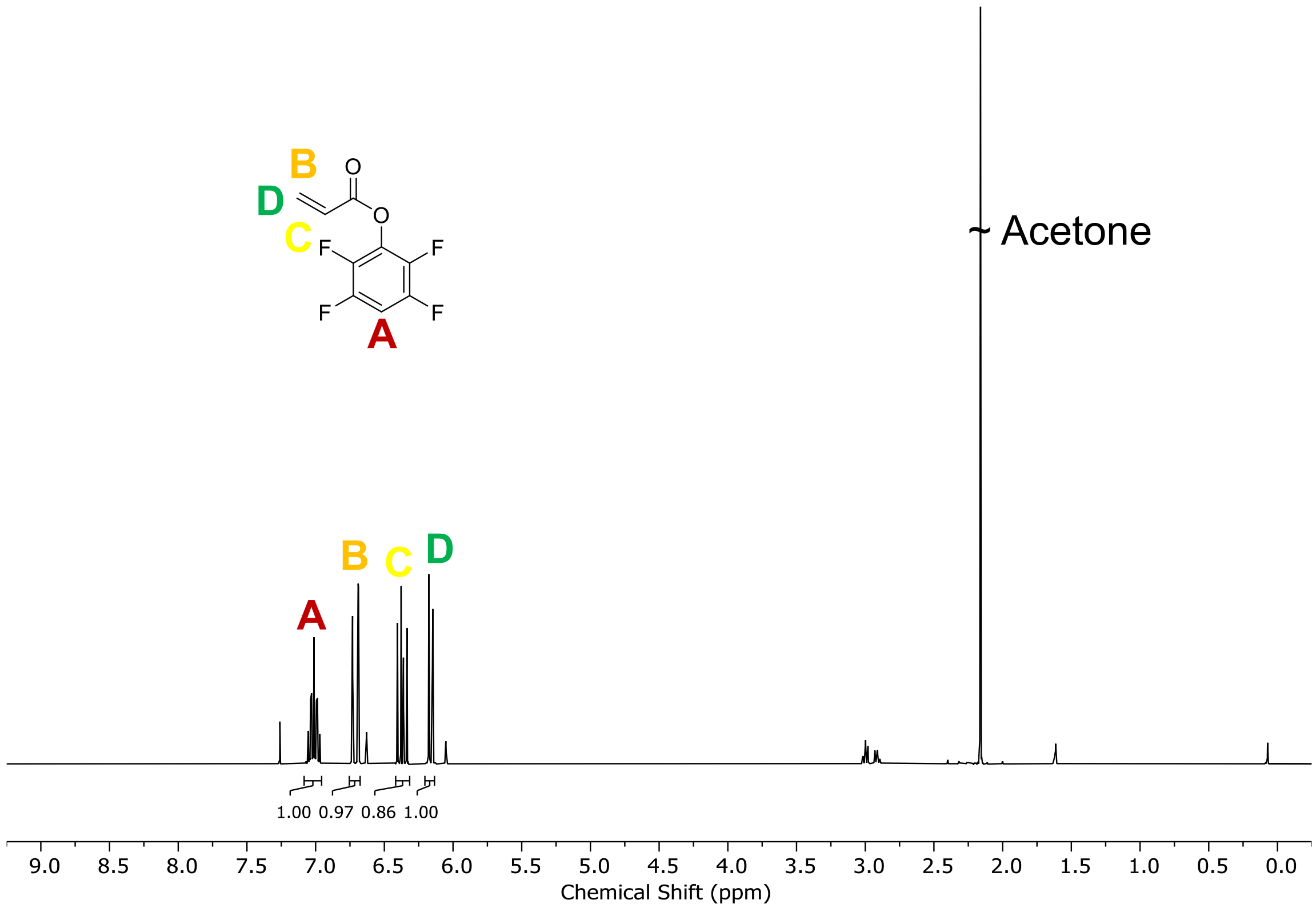

Figure S1. *^{1}H NMR (400 MHz) spectrum of tetrafluorophenyl acrylate (TFPA) in $CDCl_3$.*

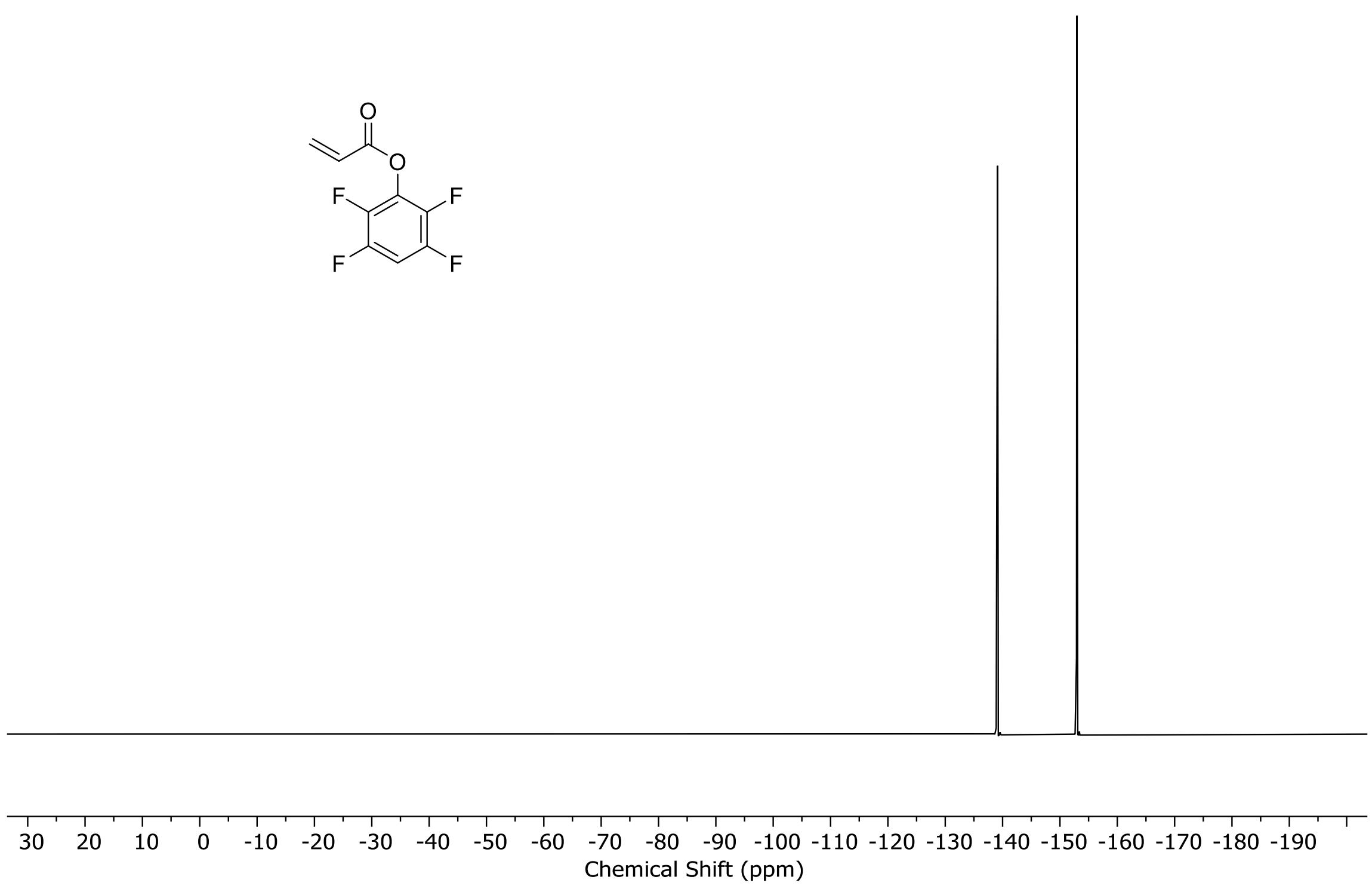

Figure S2. *^{19}F NMR (375 MHz) spectrum of tetrafluorophenyl acrylate (TFPA) in $CDCl_3$.*

Synthesis of 2-(Dodecylthiocarbonothioylthio)-2-methylpropionic acid (DDMAT):

Caution! Carbon disulfide (CS_2) should be handled in a fume hood because it is highly volatile, flammable, and toxic. After CS_2 addition, all subsequent reagents were added in one portion to minimize exposure.

DDMAT was prepared as previously reported.[1] Dodecanethiol (5.0 mL, 20.9 mmol, 1.3 equiv.) and K_3PO_4 (3.60 g, 16.9 mmol, 1.1 equiv.) were combined in a 100 mL round bottom flask with 70 mL of acetone. A magnetic stir bar was added and the solution was allowed to mix for 20 minutes before CS_2 (3.0 mL, 49.6 mmol, 3.1 equiv.) was added dropwise using a syringe. To the now yellow solution, 2-bromo-2-methyl-propionic acid (2.64 g, 15.8 mmol, 1 equiv.) was added in one portion and allowed to react at room temperature for 15 h. The solution was added to a separatory funnel along with ~200 mL of 3 M HCl. The mixture was extracted twice with 200 mL of dichloromethane (DCM), then the combined organic layers were washed with ~150 mL of brine, then dried over $MgSO_4$. The DCM was removed under reduced pressure, then the crude oil was doused with hexanes and placed back on the rotary evaporator, at which point yellow crystals began to form. The product was recrystallized from hot hexanes, washed with 0.5 M HCl, and dried under reduced pressure (4.52g, 78% yield).

^1H NMR (400 MHz, $CDCl_3$, δ): 3.3 (t, 2H), 1.7 (m, 8H), 1.4 (m, 2H), 1.3 (s, 16H), 0.9 (t, 3H)

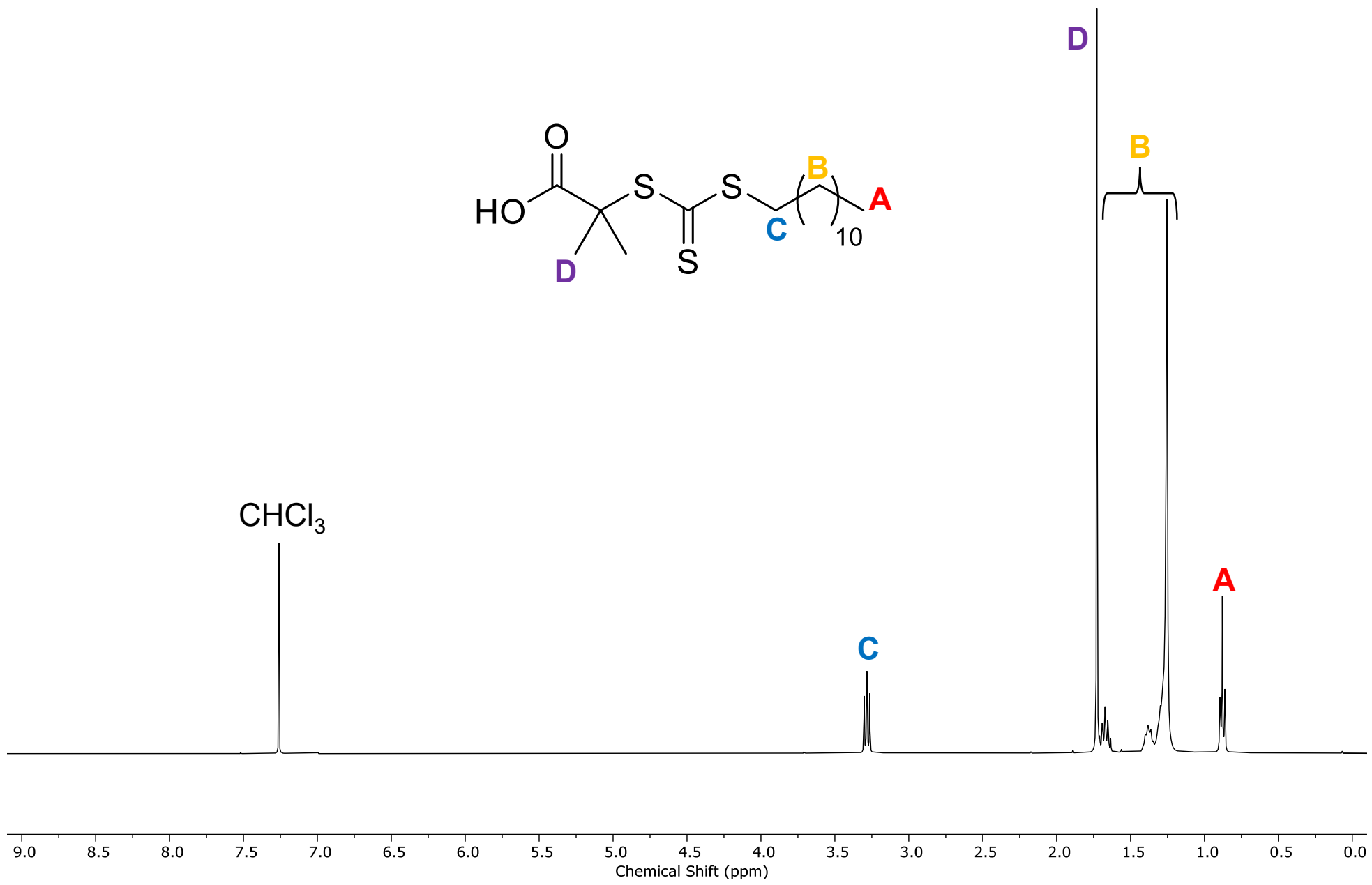

Figure S3. *^{1}H NMR (400 MHz) spectrum of DDMAT in $CDCl_3$.*

RAFT Polymerization of TFPA:

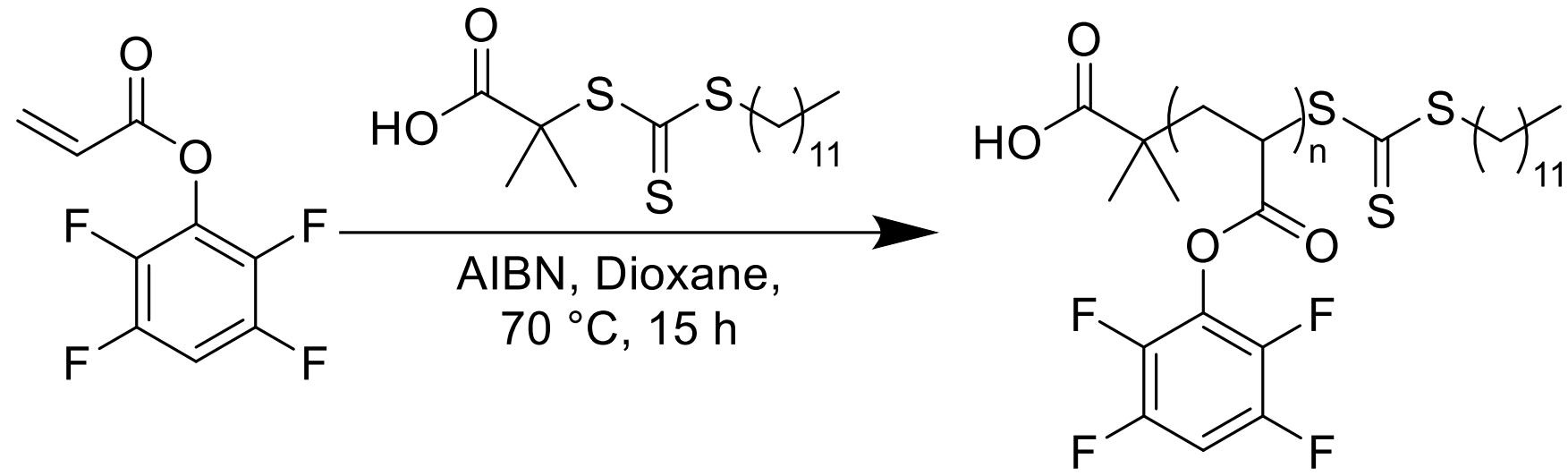

DDMAT (0.1658g, 0.454 mmol, 1 equiv.), TFPA (10.0g, 45.4 mmol, 100 equiv.), and AIBN (7.5 mg, 0.045 mmol, 0.1 equiv.) were combined in a glass vial with 7 mL of 1,4-dioxane. A small crystal of 1,3,5-trioxane was added as an internal NMR standard and an initial NMR aliquot was taken. The vial was sealed, then sparged with N_2 for 30 minutes before being added to a heating block equilibrated at 70 °C and allowed to react for 5.5 hours. The viscous reaction solution was then exposed to air and a crude NMR sample was taken. ^{1}H NMR of the crude sample indicated a 93% conversion. The solvent was removed *in vacuo,* then the sample was redissolved in THF (~7 mL) and precipitated into chilled methanol (~200 mL). The precipitate was filtered then the polymer re-precipitated twice more before being dried at room temperature on a Schlenk line vacuum overnight. ^{1}H NMR end group analysis and size exclusion chromatography (SEC) were used to characterize the polymer molecular weight (**Figures S4 & S6**).

^{1}H NMR (400 MHz, $CDCl_3$, δ): 6.9 (br s), 3.2 (br s), 2.6-1.9 (br m), 0.9 (t, 3H); M_n = 22.9 kDa (end-group analysis)

^{19}F NMR (375 MHz, $CDCl_3$, δ): -139 (br s), -153 (br s).

SEC (MALS, DMAc w/ 50 mM LiCl, *dn/dc*=0.0477): M_n = 24.7 kDa*,* $Đ$ = 1.43

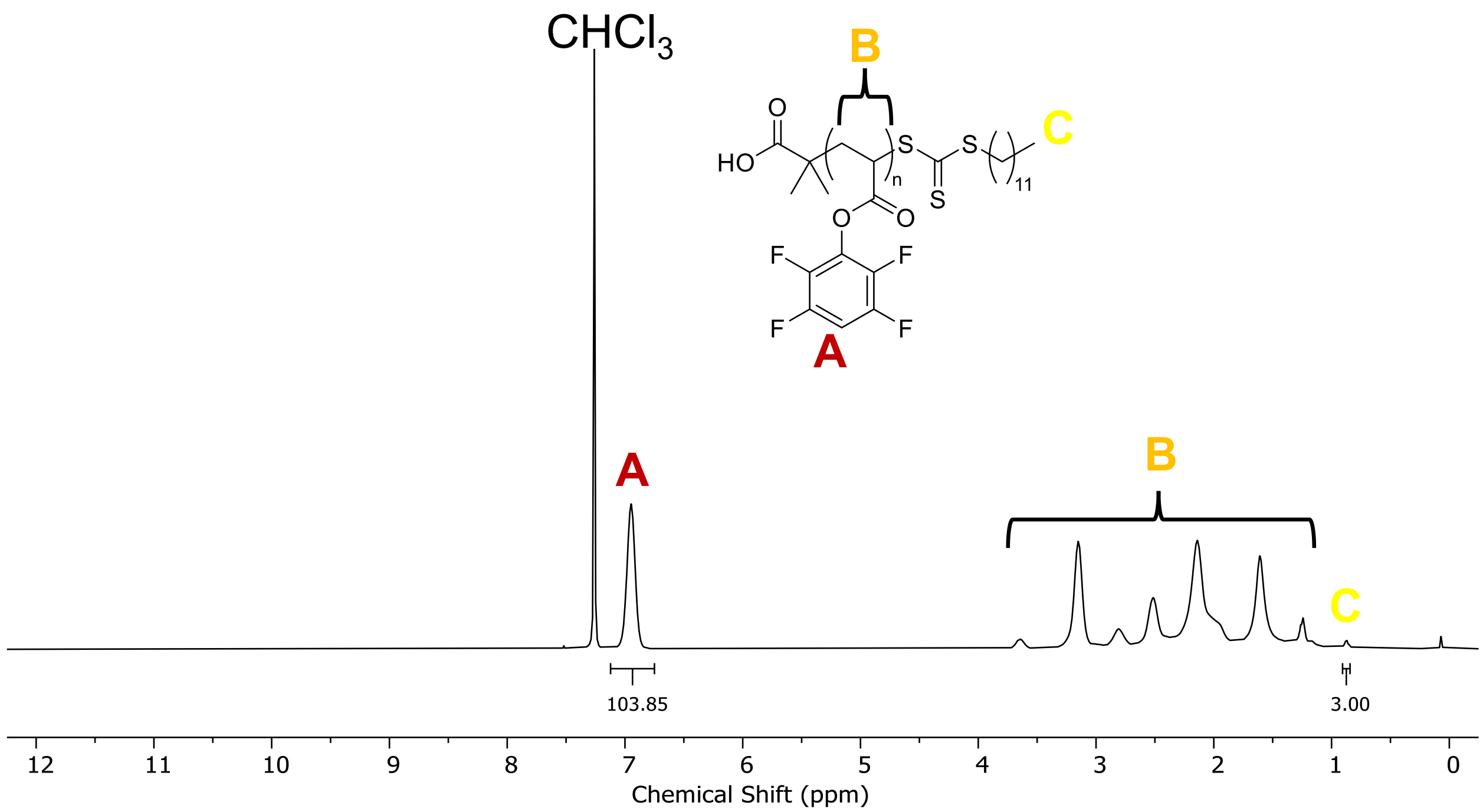

Figure S4. *^{1}H NMR (400 MHz) spectrum of poly(tetrafluorophenyl acrylate) (PTFPA) in $CDCl_3$.*

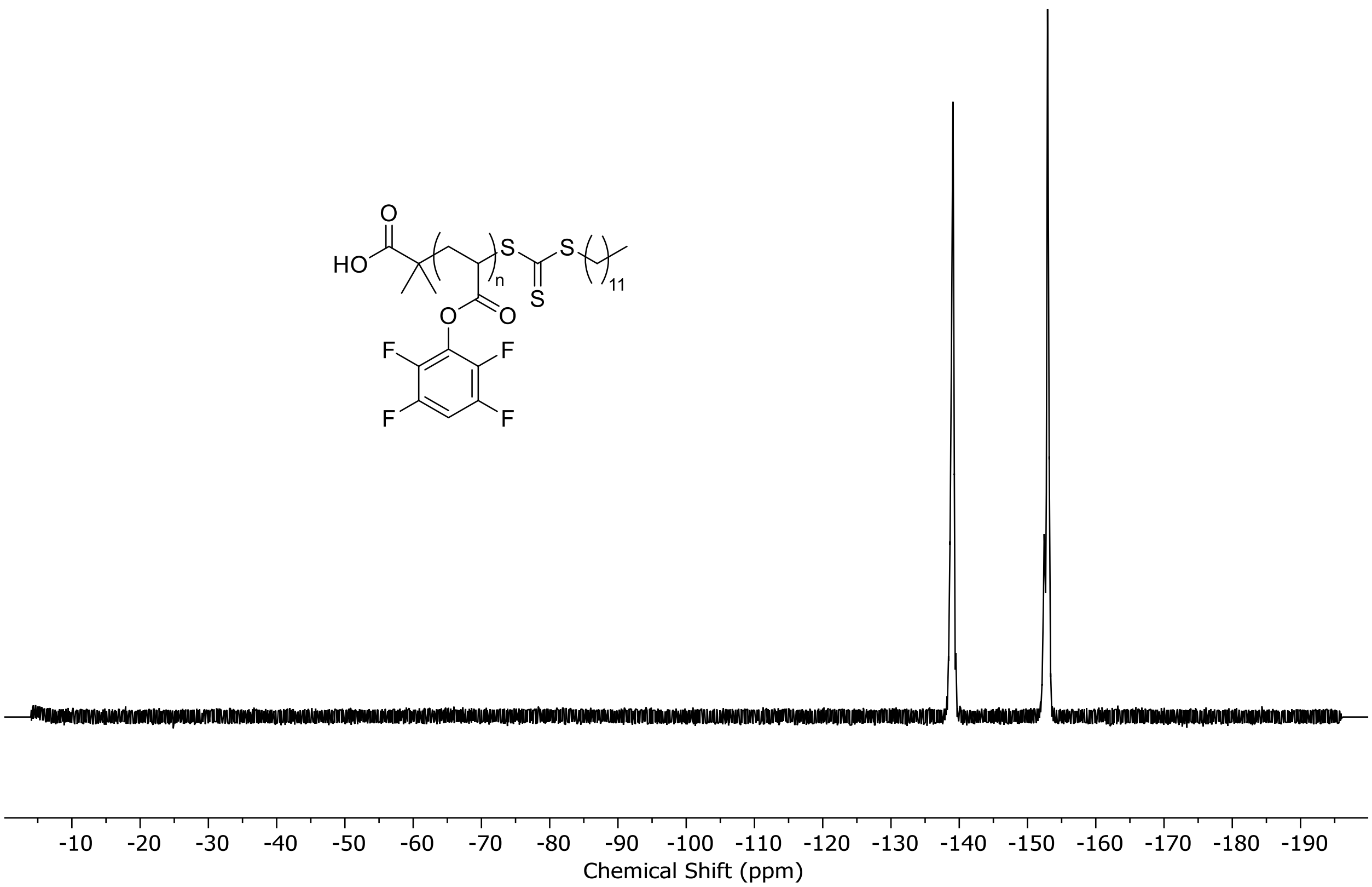

Figure S5. *^{19}F NMR (375 MHz) spectrum of poly(tetrafluorophenyl acrylate) (PTFPA) in CDCl₃.*

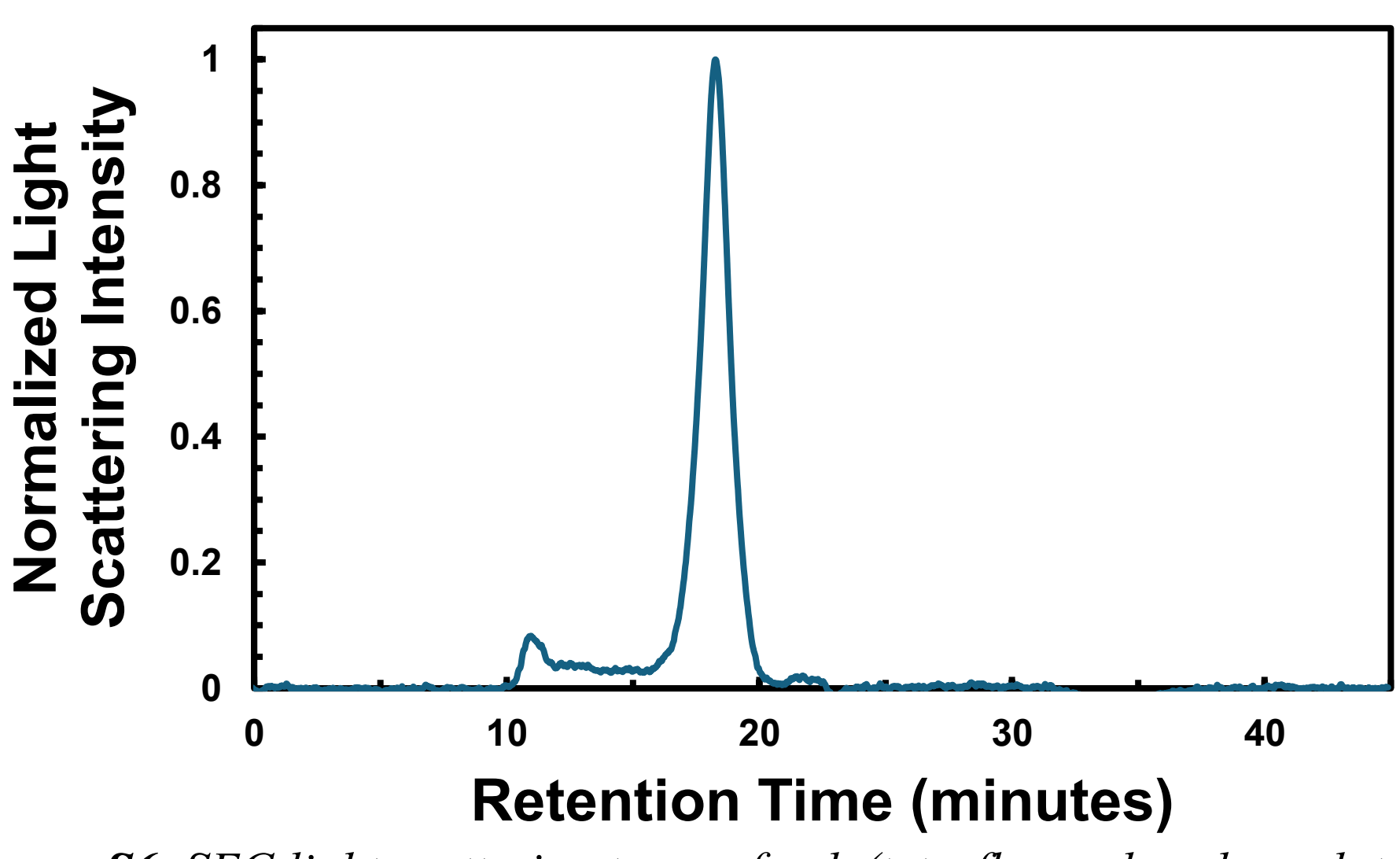

Figure S6. *SEC light scattering trace of poly(tetrafluorophenyl acrylate) (PTFPA) in DMAc with 50 mM LiCl.*

Representative Post-Polymerization Modification (poly(aspartic acid acrylamide)):

PTFPA (0.1025g, 0.45 mmol, 1.0 equiv.) was placed in a 6-dram glass vial with a magnetic stir bar and dissolved in 4 mL of DMF. Triethylamine (0.069g, 0.682 mmol, 1.5 equiv.) was added followed by aspartic acid (0.0915g, 0.687 mmol, 1.5 equiv.). The reaction was added to a heating block pre-heated to 70 °C and stirred overnight for 15 hours. The vial was removed from the heat, and an aliquot was taken for ^{19}F NMR analysis. The solvent was removed *in vacuo,* and the sample was redissolved in DI H_2O before being placed in a dialysis bag and dialyzed against DI H_2O for 3 days. ^{19}F NMR confirmed full removal of the tetrafluorophenoxide leaving group while ^{1}H NMR was used to characterize the final polymer structure.

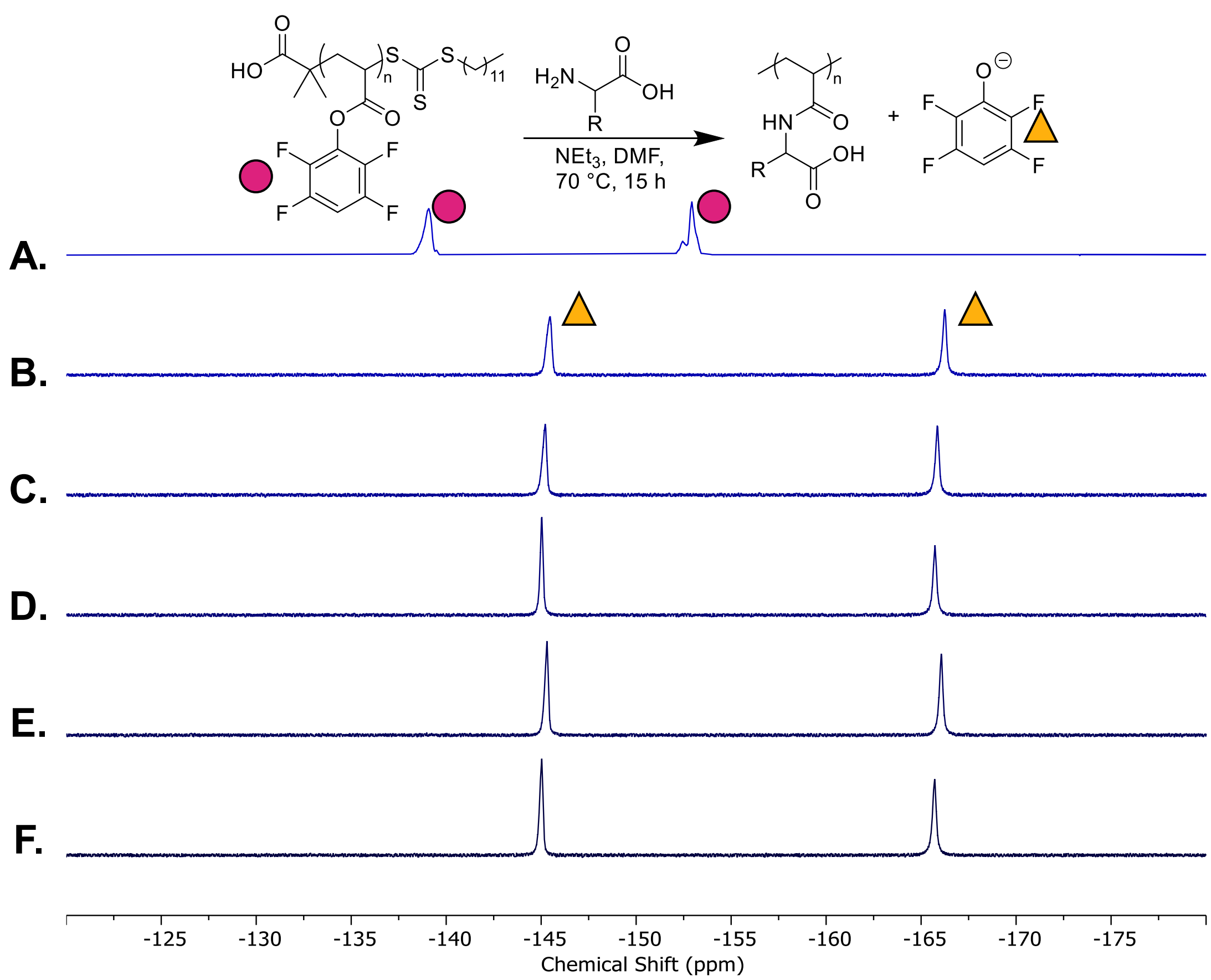

Figure S7. *^{19}F NMR (375 MHz, DMSO-d_6) spectra of PTFPA before (A) and after functionalization by glutamine (B), asparagine (C), glutamic acid (D), histidine (E), and aspartic acid (F) showing full displacement of the tetrafluorophenoxide leaving group.*

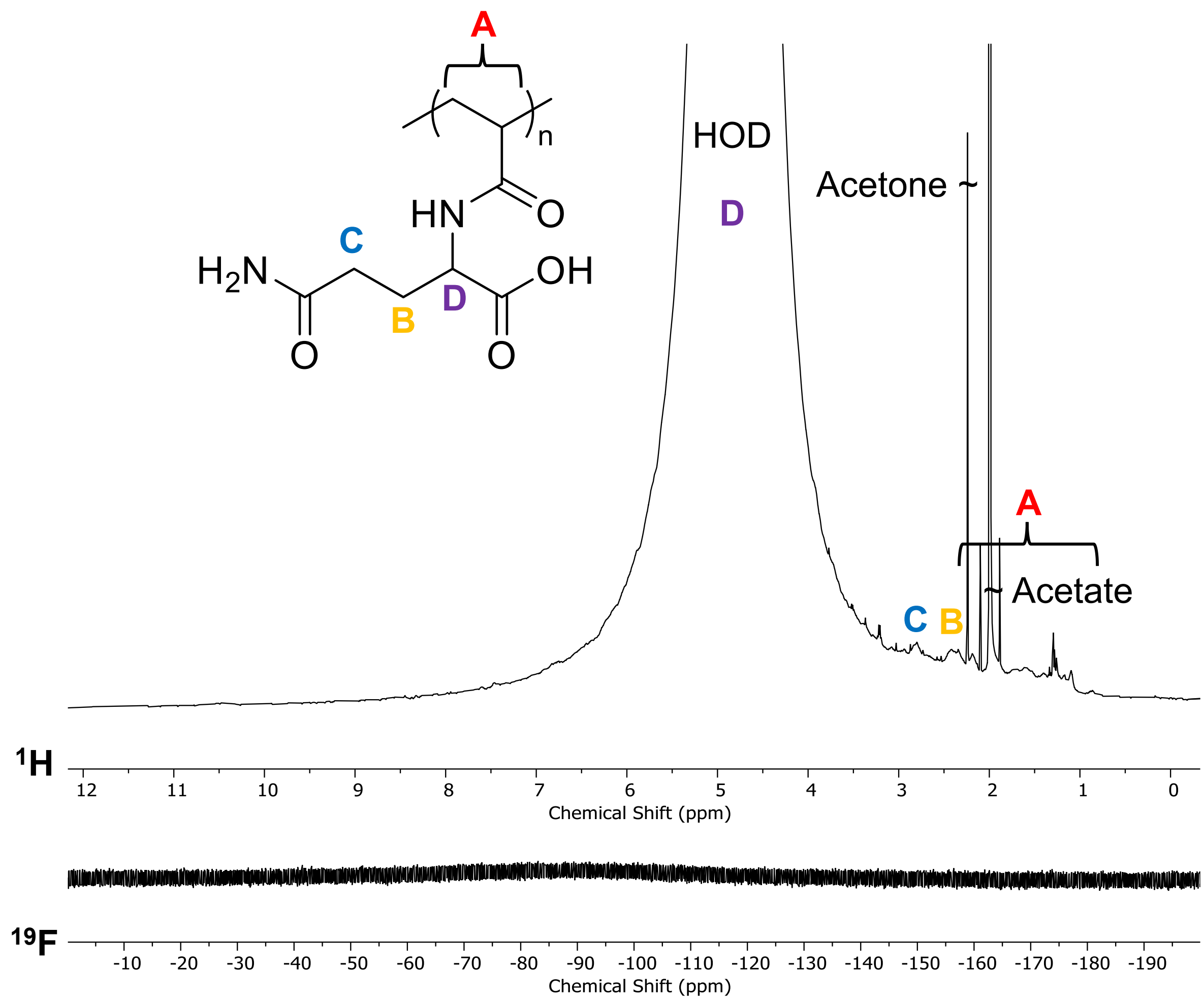

Figure S8. *^{1}H (600 MHz, D_2O, above) and ^{19}F NMR (375 MHz, D_2O, below) spectra of poly(glutamine acrylamide) in pH 5, 100 mM acetate buffer.*

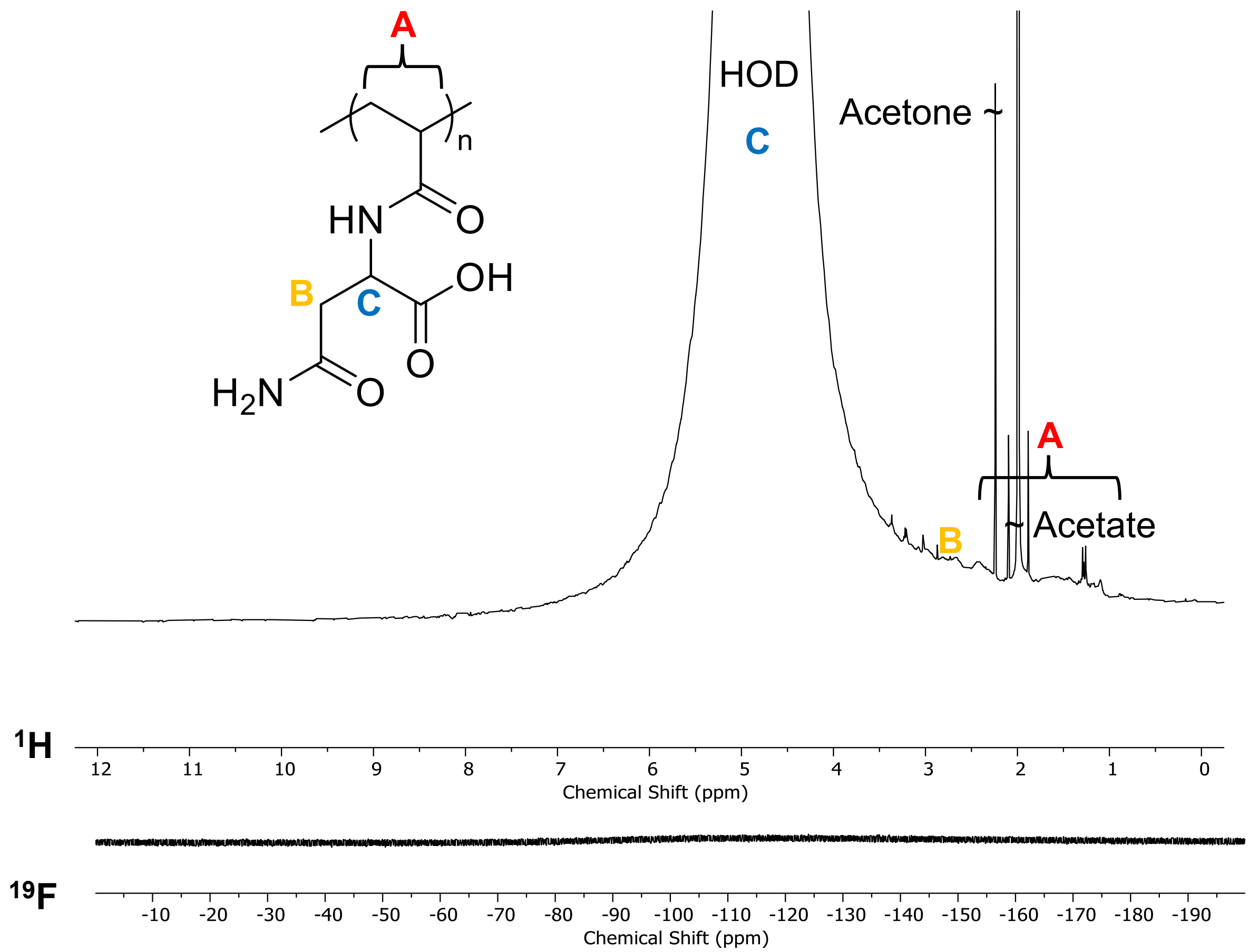

Figure S9. *^1H (600 MHz, D_2O, above) and ^{19}F (375 MHz, D_2O, below) NMR spectra of poly(asparagine acrylamide) in pH 5, 100 mM acetate buffer.*

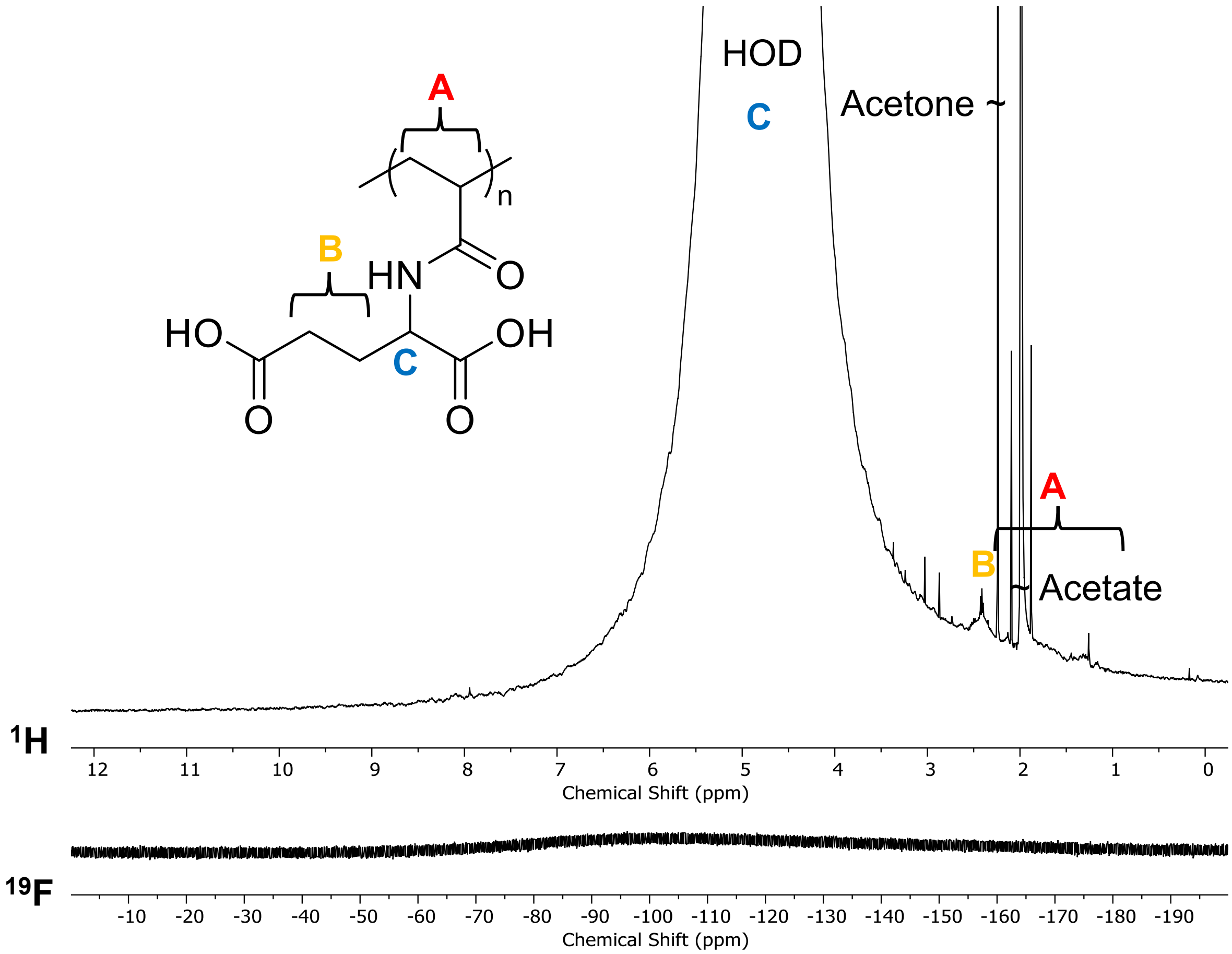

Figure S10. *^{1}H (600 MHz, D_2O, above) and ^{19}F (375 MHz, D_2O, below) NMR spectra of poly(glutamic acid acrylamide) in pH 5, 100 mM acetate buffer.*

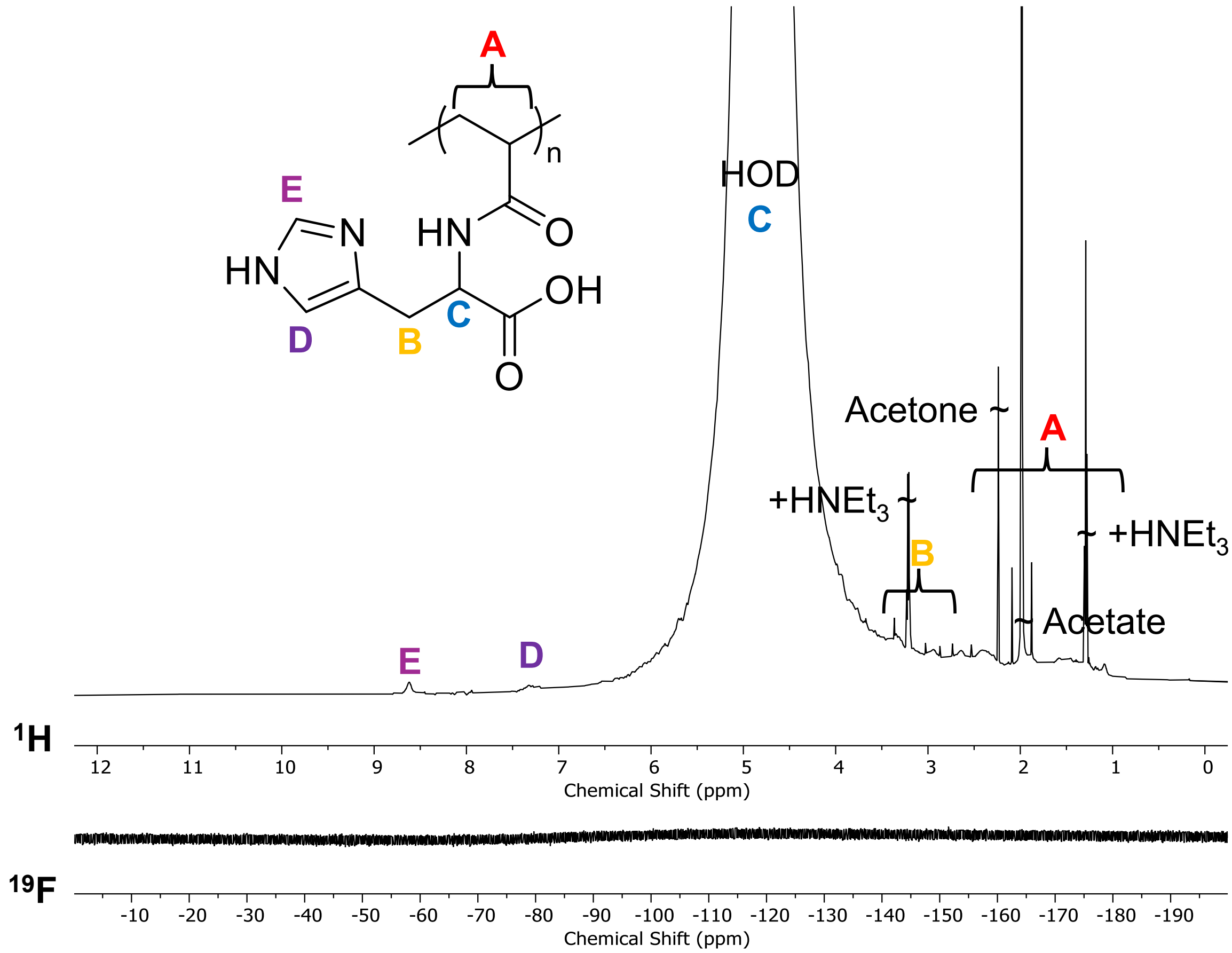

Figure S11. *^{1}H (600 MHz, D_2O, above) and ^{19}F (375 MHz, D_2O, below) NMR spectra of poly(histidine acrylamide) in pH 5, 100 mM acetate buffer.*

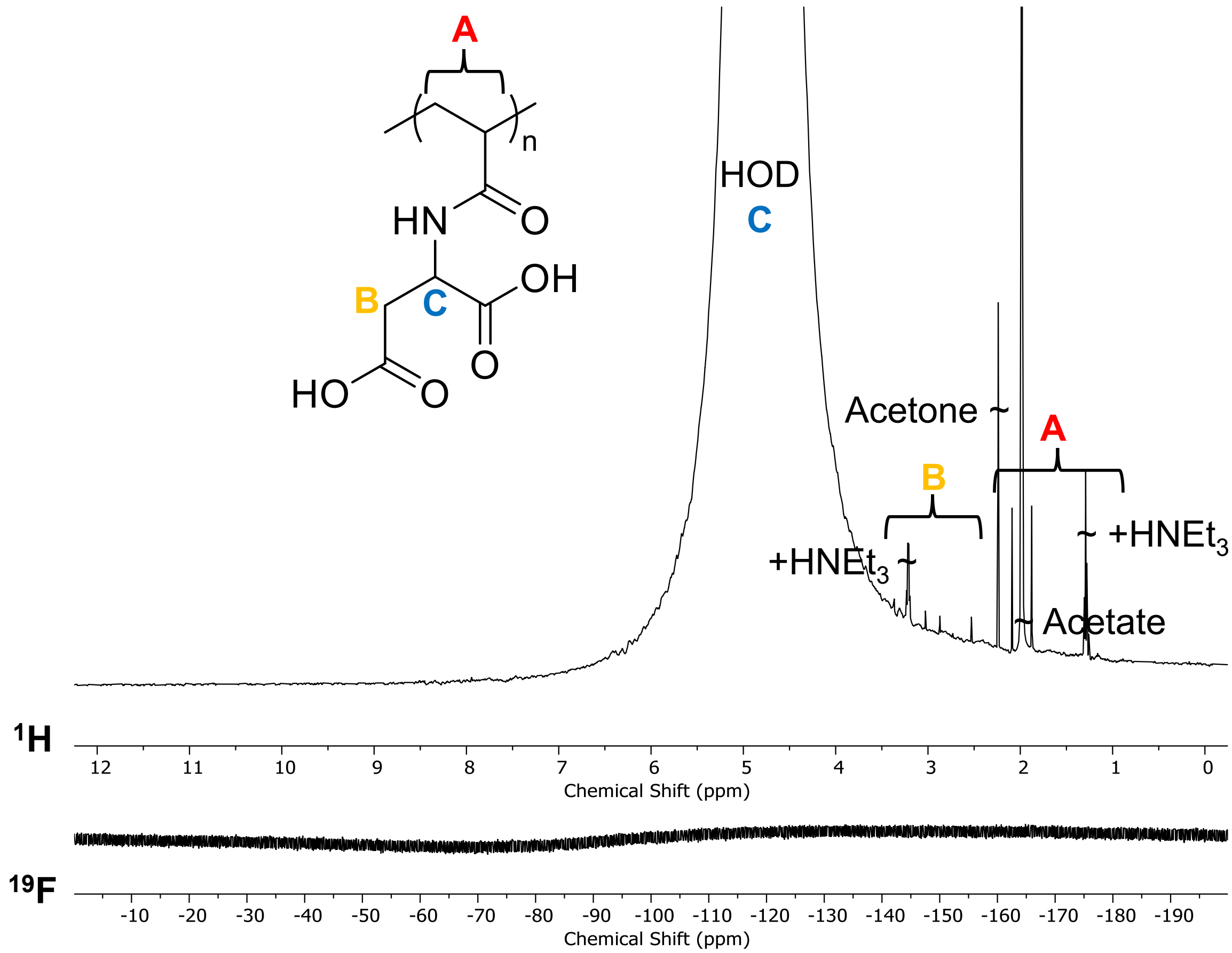

Figure S12. *^{1}H (600 MHz, D_2O, above) and ^{19}F NMR (375 MHz, D_2O, below) spectra of poly(aspartic acid acrylamide) in pH 5, 100 mM acetate buffer.*

Isothermal Titration Calorimetry

The ITC instrument used was an Affinity-ITC (TA Instruments, New Castle, DE, USA), which operated at constant temperature (278.15, 288.15, 298.15, or 308.15 K). To carry out a binding experiment, stock solutions were prepared by dissolving the polymer or $EuCl_3$ in buffer (100 mM NaOAc/HOAc, pH 5). The sample solutions were then prepared by dilution by mass of the stock solution with buffer to the desired concentration (Table S1). All polymer concentrations are given as the concentration of repeat unit, not the entire polymer chain.

Before the ITC experiment, each solution was degassed for 15 min in a TA Instruments Degassing Station Model 6326 at 25 °C and 570 mmHg. The polymer solution (350 µL) was placed in the sample cell and the reference cell was filled with degassed deionized H_2O (300 µL). Both cells were mounted in an adiabatic chamber operated at a constant temperature during the measurement. A long-needle syringe with a twisted paddle fastened to its end, was filled with the metal-ion solution. The syringe was placed inside the sample cell, and the entire syringe assembly was rotated continuously (125 RPM) to provide proper mixing of the contents of the sample cell within a few seconds after an injection.

After reaching thermal equilibrium, the injection of the metal ion solution was automatically conducted stepwise until the polymer was saturated with metal ion. Due to the possible dilution of the first measurement, the first injection was ignored in the data analysis, which is standard practice for ITC experiments.[2,3] The first 1.0 µL injection was followed by a series of 24, 2.0 µL injections. The time interval between two consecutive injections was 200 – 300 s to ensure that the equilibrium was reached before the next injection.

Nonlinear least-squares curve fitting was conducted with the TA Instruments Nano Analyze program and used the TA Instruments Independent model. Heats of dilution from control titrations

(metal ion into buffer (no polymer present), and buffer into polymer (without metal ion present)) were subtracted before curve fitting using the point-by-point method. Each ITC experiment was conducted in duplicate and thermodynamic parameters are reported as the average with error reported as one standard deviation. The sigmoidal shape of the titration curve is most consistent with the "independent model" (Equation S1) corresponding to *n* identical binding sites.[2,4]

$$\frac{dQ}{d[X]_t} = H^o V_o \left[\frac{1}{2} + \frac{1 - X_R - \frac{1}{c}}{2\sqrt{(1 + X_r + \frac{1}{c})^2 - 4X_R}} \right] \quad \text{(S1)}$$

This equation relates the change in heat normalized to moles of titrant (Eu^{3+} ions in this case) added per injection ($\frac{dQ}{d[X]_t}$) to the ratio of titrant to titrate (X_R) over the course of the experiment. The "Wiseman c value" (c) is the product of the binding affinity and the receptor concentration, and V_0 is the effective cell volume of the calorimeter.

Table S1. *Optimized ITC concentrations. Polymer concentrations are given as repeat unit concentrations.*

**Amino Acid Side Chain**	**[Polymer] (mM)**	**[Eu^{3+}] (mM)**
Glutamine	3.00	3.51
Asparagine	3.00	3.51
Glutamic Acid	3.00	2.00
Histidine	2.24	2.70
Aspartic Acid	1.50	2.26

Table S2. *Thermodynamics of Eu^{3+} binding by hydrophilic poly(amino acid acrylamides)*

**Material**	**Temperature (°C)**	**Log (K_a)**	**Repeat units per Eu(III)**	**ΔG (kJ mol^{-1})**	**ΔH (kJ mol^{-1})**	**-TΔS (kJ mol^{-1})**	**ΔC$_P$ (kJ mol^{-1} K^{-1})**
Glutamine	5	4.27 ± 0.01	5.5 ± 0.3	-22.73 ± 0.07	6.61 ± 0.01	-29.34 ± 0.07	0.166 ± 0.009
	15	4.24 ± 0.03	5.48 ± 0.06	-23.4 ± 0.2	9.2 ± 0.1	-32.60 ± 0.05	
	25	4.43 ± 0.07	5.06 ± 0.09	-25.3 ± 0.4	10.3 ± 0.3	-35.6 ± 0.1	
	35	4.63 ± 0.05	5.3 ± 0.1	-27.3 ± 0.3	11.7 ± 0.2	-39.0 ± 0.1	
Asparagine	5	4.9 ± 0.1	3.68 ± 0.03	-26.0 ± 0.6	5.81 ± 0.08	-31.8 ± 0.5	0.199 ± 0.002
	15	4.66 ± 0.02	3.95 ± 0.07	-25.7 ± 0.1	8.5 ± 0.1	-34.16 ± 0.01	
	25	4.7 ± 0.1	4.02 ± 0.01	-26.5 ± 0.5	10.12 ± 0.04	-36.6 ± 0.5	
	35	4.93 ± 0.06	4.2 ± 0.1	-29.1 ± 0.4	11.9 ± 0.1	-41.0 ± 0.3	
Glutamic Acid	5	4.18 ± 0.04	11.3 ± 0.3	-22.3 ± 0.2	11.4 ± 0.4	-33.6 ± 0.2	0.09 ± 0.04
	15	4.42 ± 0.04	12.7 ± 0.1	-24.4 ± 0.2	12.7 ± 0.5	-37.1 ± 0.2	
	25	4.58 ± 0.05	11.8 ± 0.3	-26.2 ± 0.3	13.3 ± 0.5	-39.5 ± 0.2	
	35	4.87 ± 0.06	12.2 ± 0.4	-28.7 ± 0.4	14.1 ± 0.4	-42.8 ± 0.8	
Histidine	5	4.39 ± 0.08	5.5 ± 0.4	-23.4 ± 0.4	10.7 ± 0.5	-34.10 ± 0.08	0.18 ± 0.01
	15	4.50 ± 0.01	5.57 ± 0.07	-24.80 ± 0.02	13.5 ± 0.1	-38.29 ± 0.08	
	25	4.6 ± 0.1	5.5 ± 0.2	-26.3 ± 0.8	15.1 ± 0.8	-41.47 ± 0.06	
	35	4.9 ± 0.1	5.0 ± 0.4	-28.7 ± 0.6	16.1 ± 0.6	-44.8 ± 0.1	
Aspartic Acid	5	4.27 ± 0.05	4.3 ± 0.2	-22.7 ± 0.2	9.2 ± 0.2	-31.9 ± 0.1	0.16 ± 0.02
	15	4.50 ±0.03	4.33 ± 0.01	-24.8 ± 0.1	9.8 ± 0.4	-34.6 ± 0.2	
	25	4.51 ± 0.05	4.9 ± 0.4	-25.7 ± 0.3	11.2 ± 0.8	-36.9 ± 0.6	
	35	4.8 ± 0.1	5.1 ± 0.5	-28.3 ± 0.8	13.9 ± 0.7	-42.21 ± 0.06	

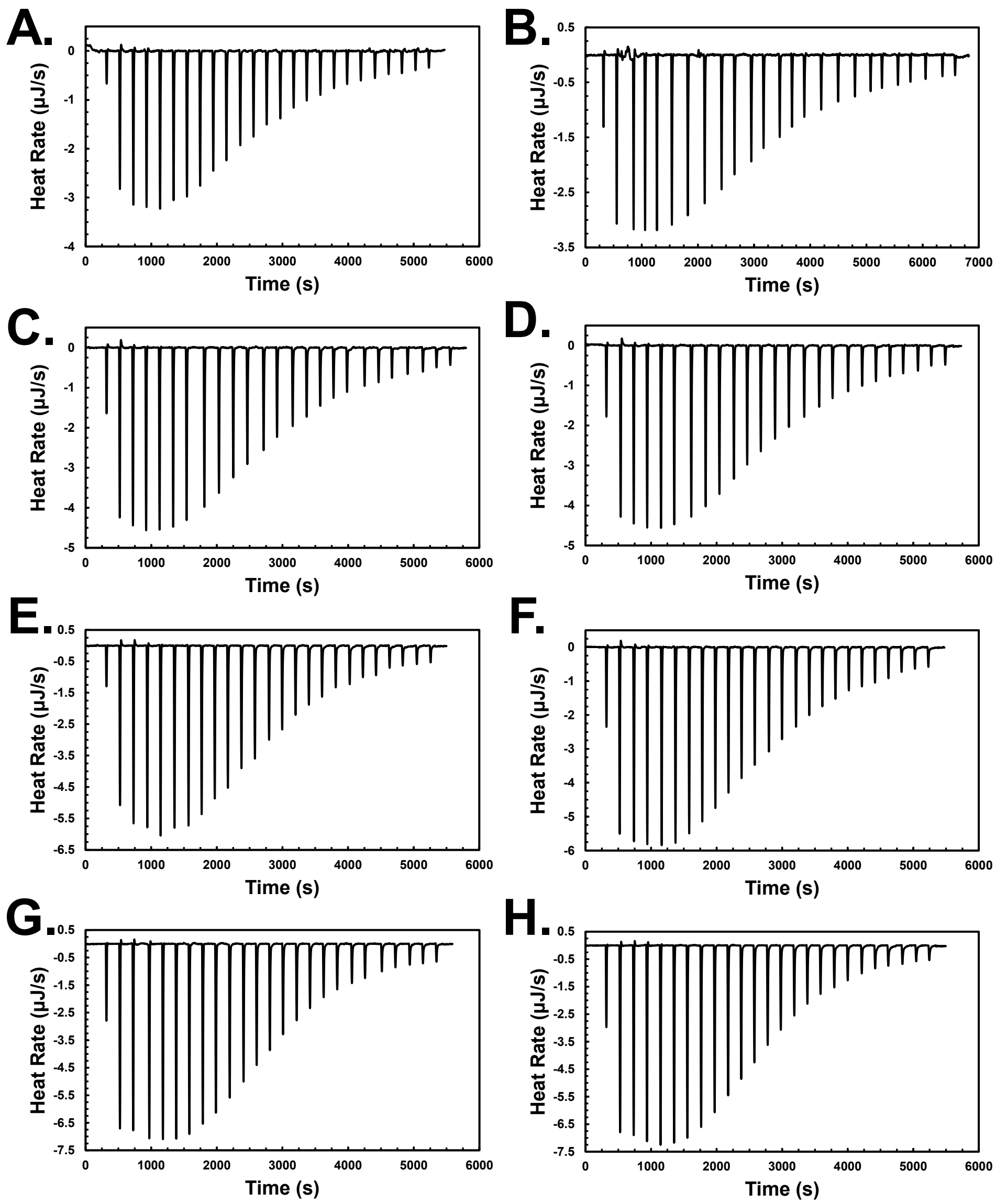

Figure S13. *ITC thermograms for Eu^{3+} binding by poly(glutamine acrylamide) at 5(A & B), 15(C & D), 25(E & F), and 35(G & H) °C.*

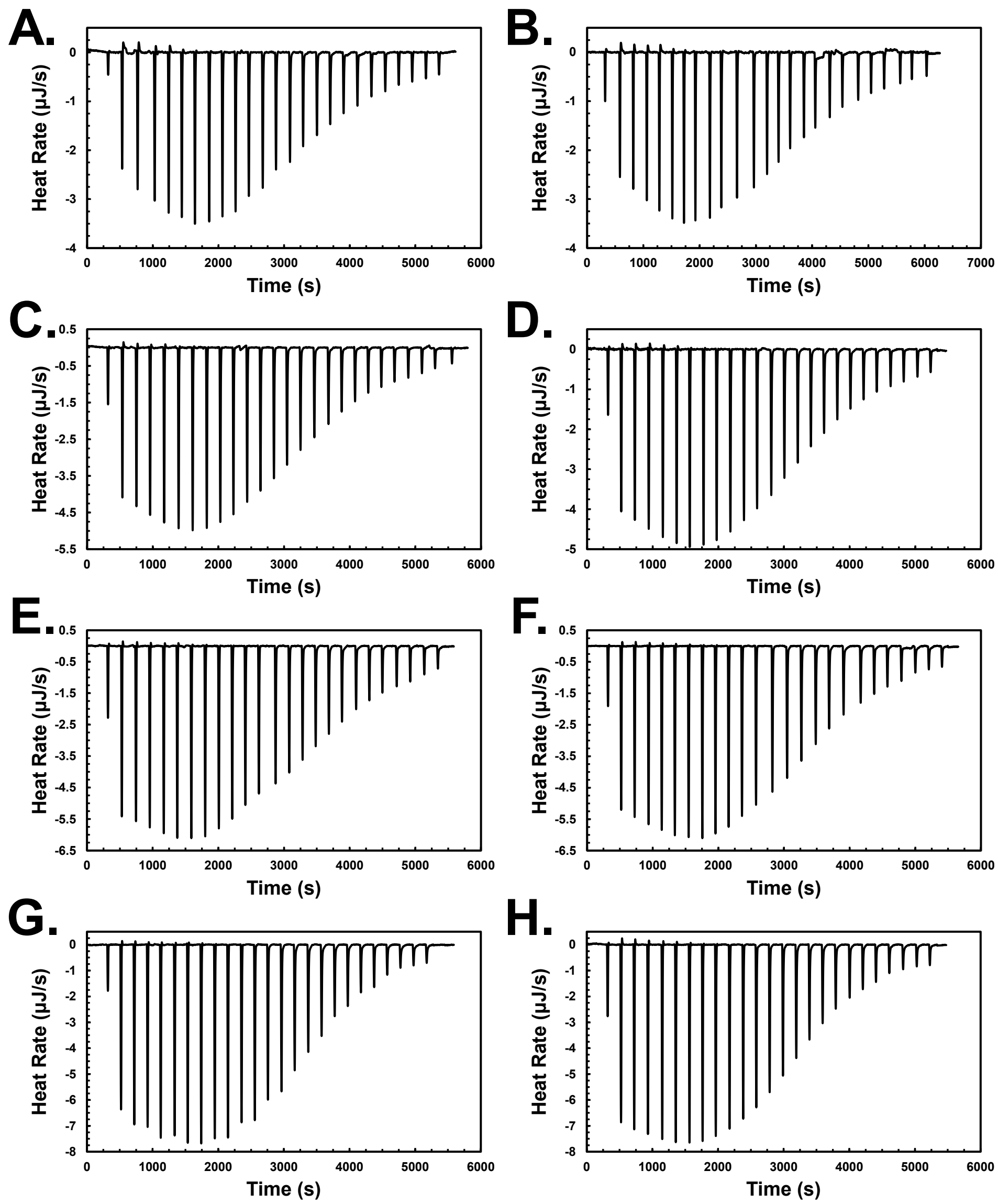

Figure S14. *ITC thermograms for Eu^{3+} binding by poly(asparagine acrylamide) at 5(A & B), 15(C & D), 25(E & F), and 35(G & H) °C.*

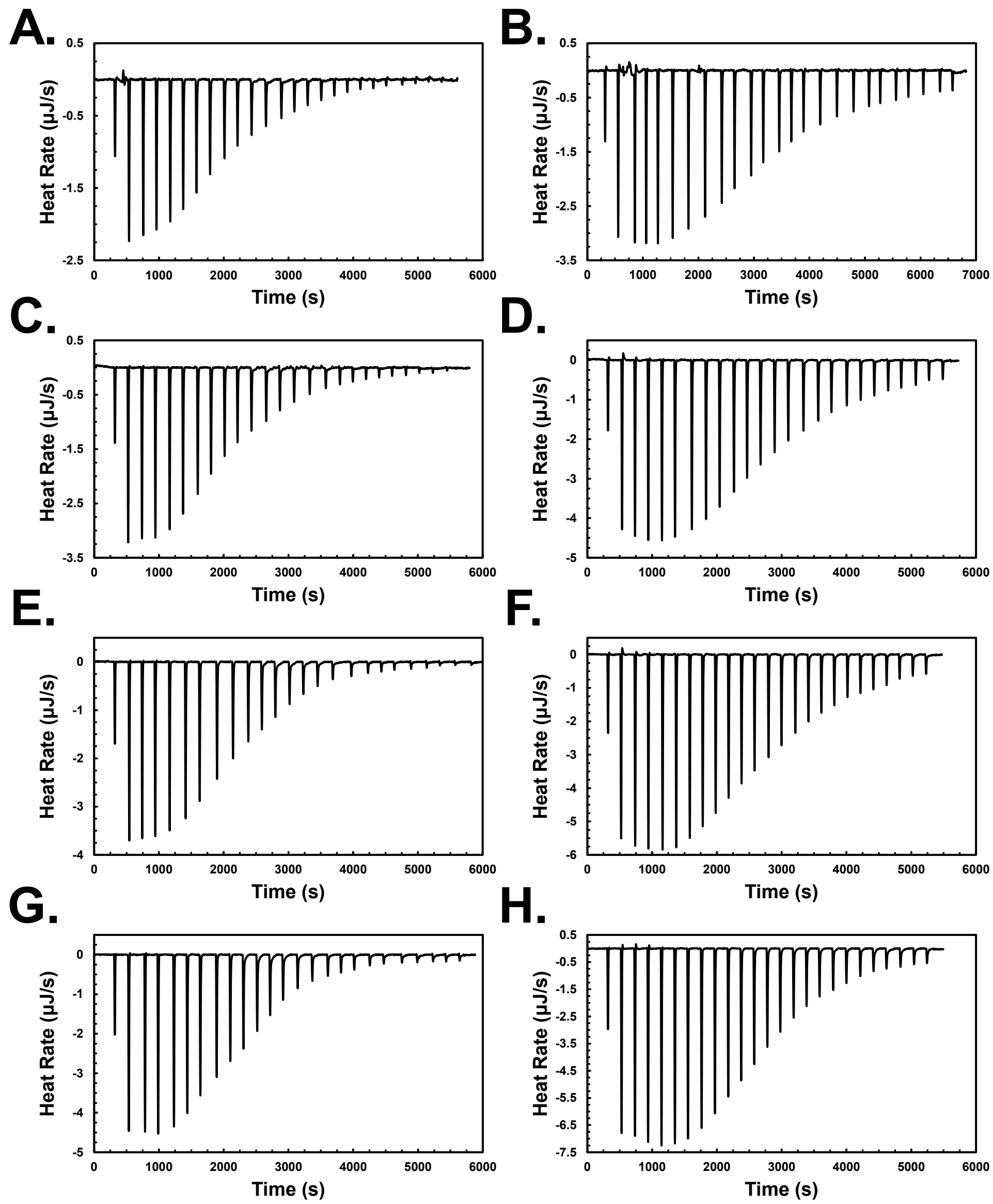

Figure S15. *ITC thermograms for Eu^{3+} binding by poly(glutamic acid acrylamide) at 5(A & B), 15(C & D), 25(E & F), and 35(G & H) °C.*

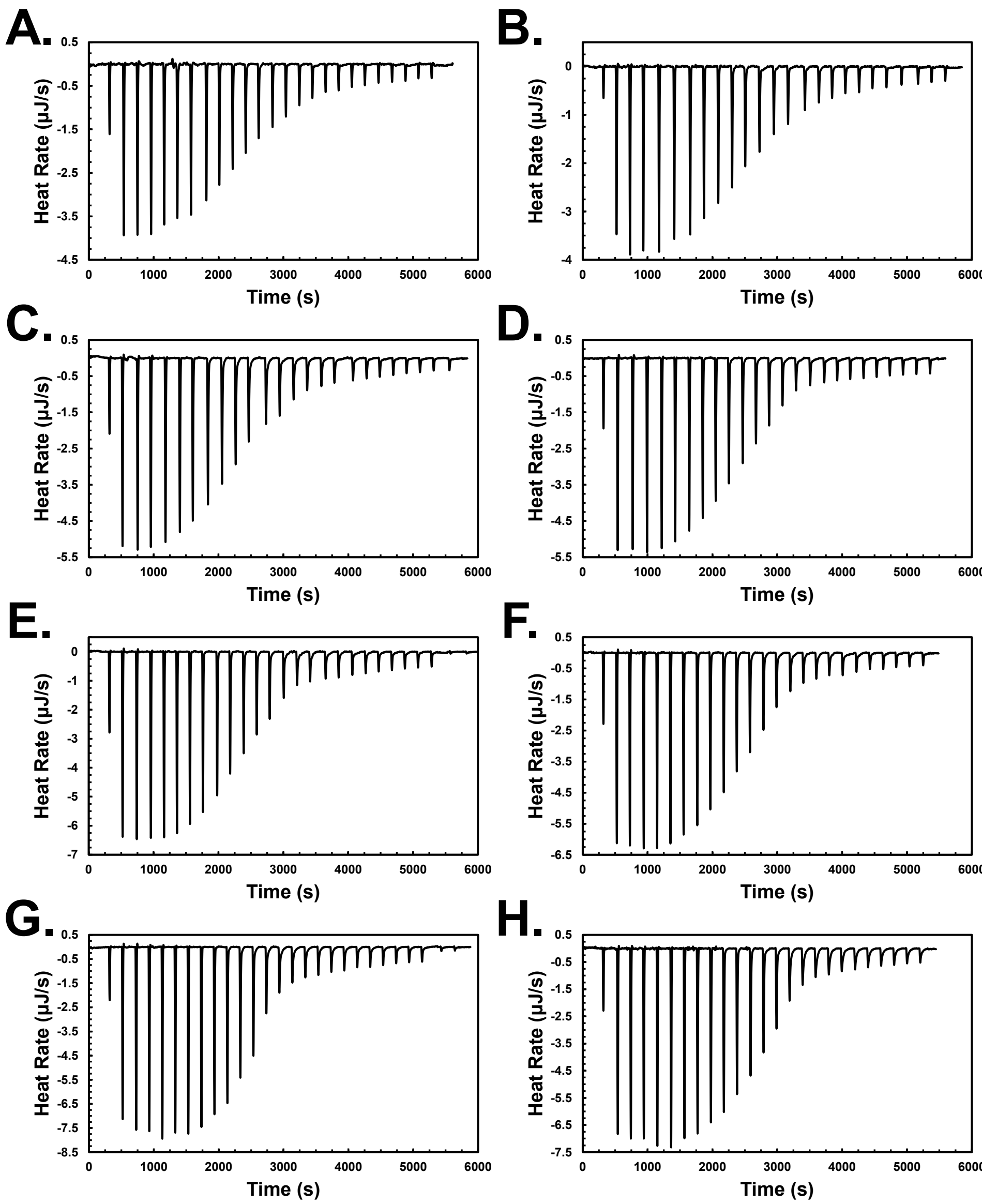

Figure S16. *ITC thermograms for Eu^{3+} binding by poly(histidine acrylamide) at 5(A & B), 15(C & D), 25(E & F), and 35(G & H) °C.*

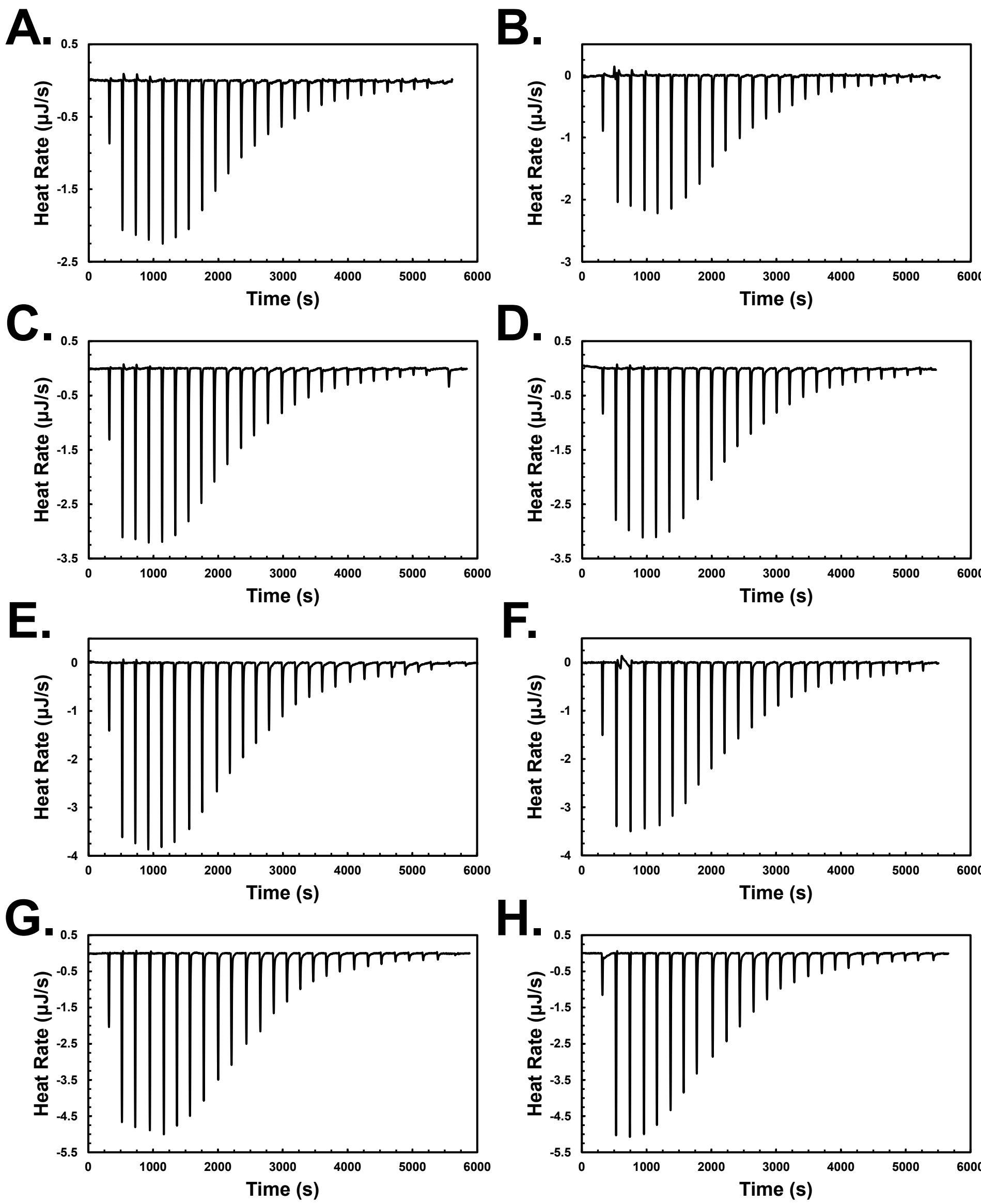

Figure S17. *ITC thermograms for Eu^{3+} binding by poly(aspartic acid acrylamide) at 5(A & B), 15(C & D), 25(E & F), and 35(G & H) °C.*

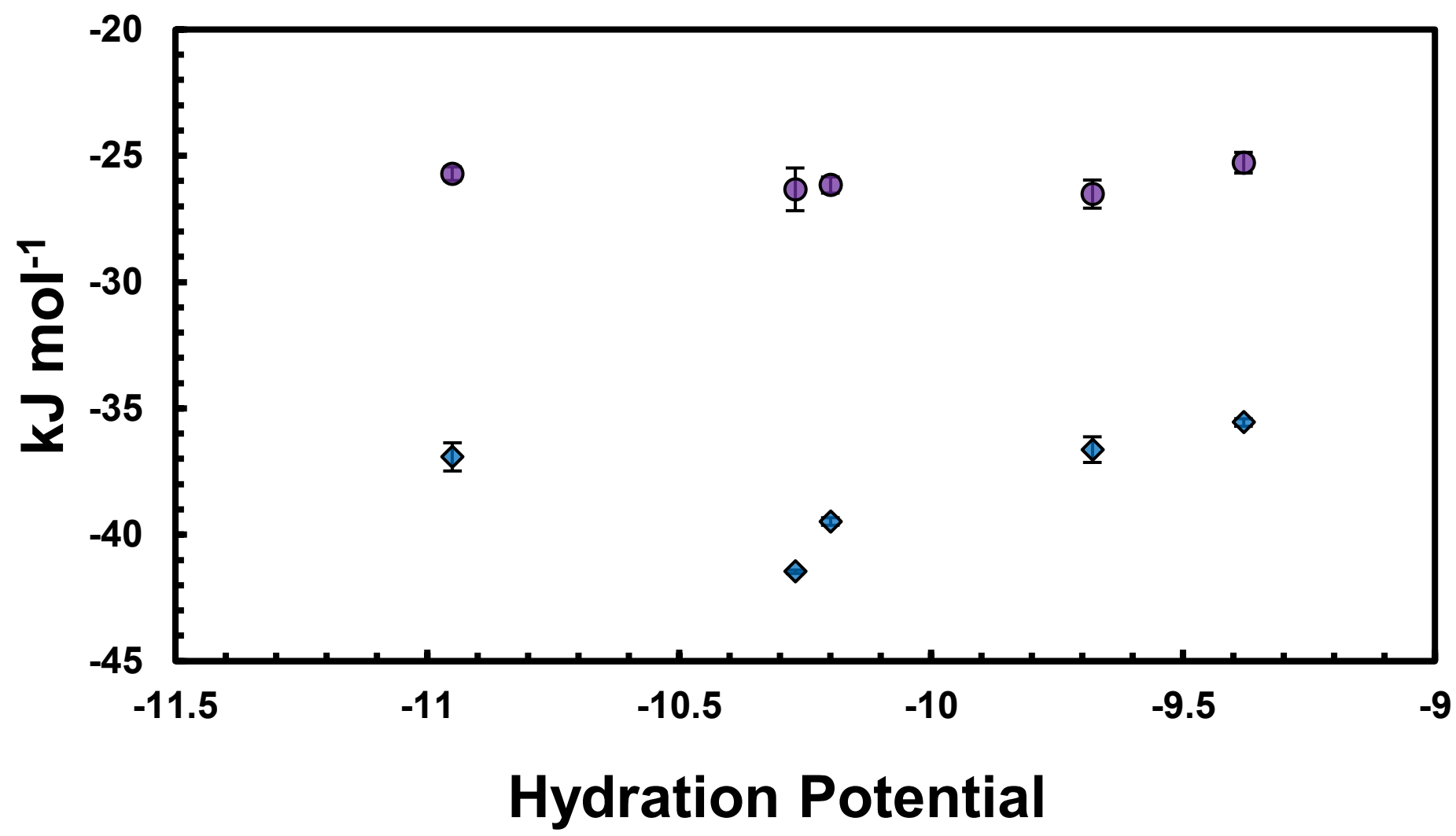

Figure S18. *ΔG (purple circles) and -TΔS (blue diamonds) for* Eu^{3+} *binding by hydrophilic poly(amino acid acrylamides) as a function of amino acid side chain hydration potential.*[5] *Error bars are one standard deviation.*

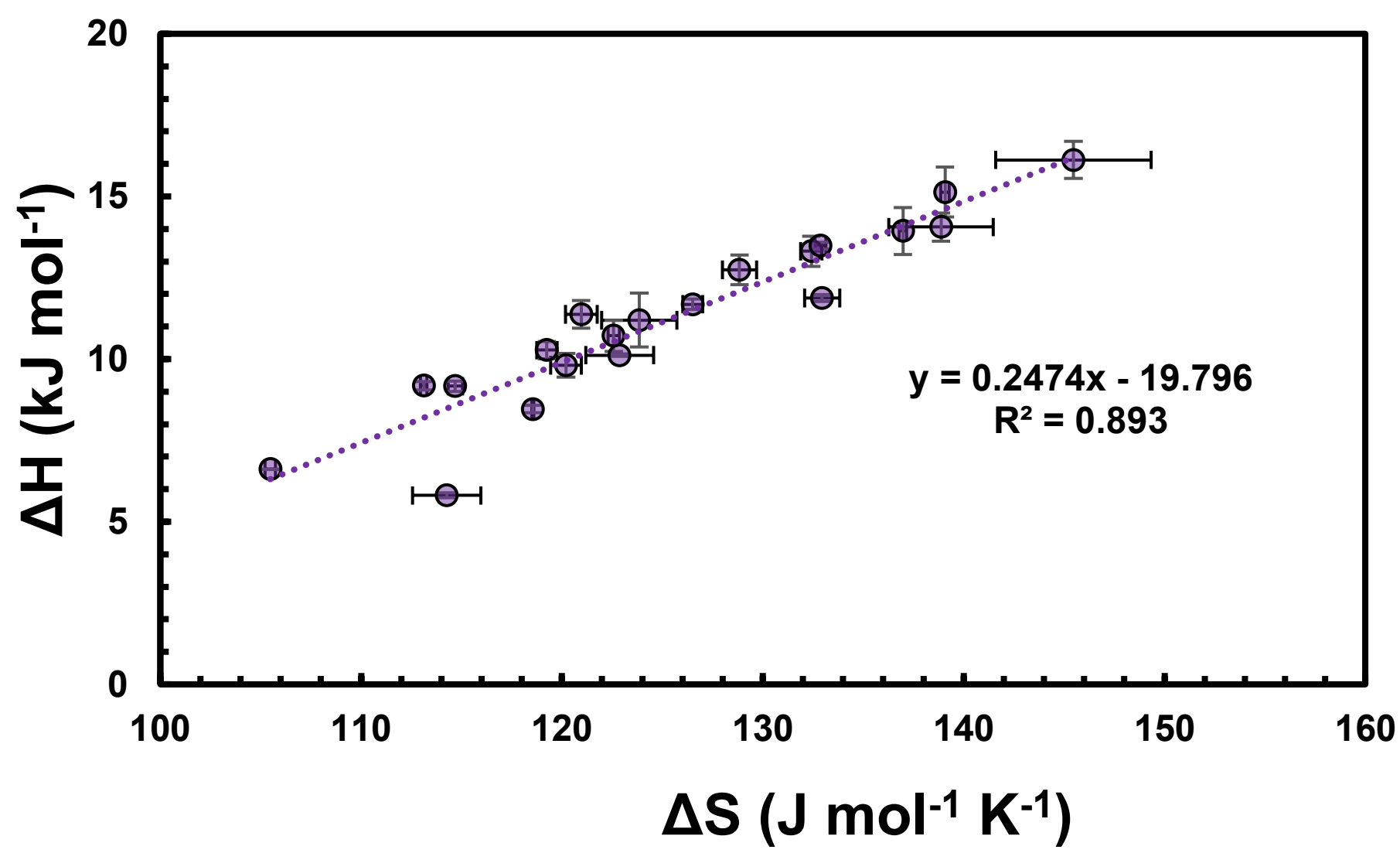

Figure S19. *ΔS vs ΔH for* Eu^{3+} *binding by hydrophilic poly(amino acid acrylamides). Error bars are one standard deviation.*

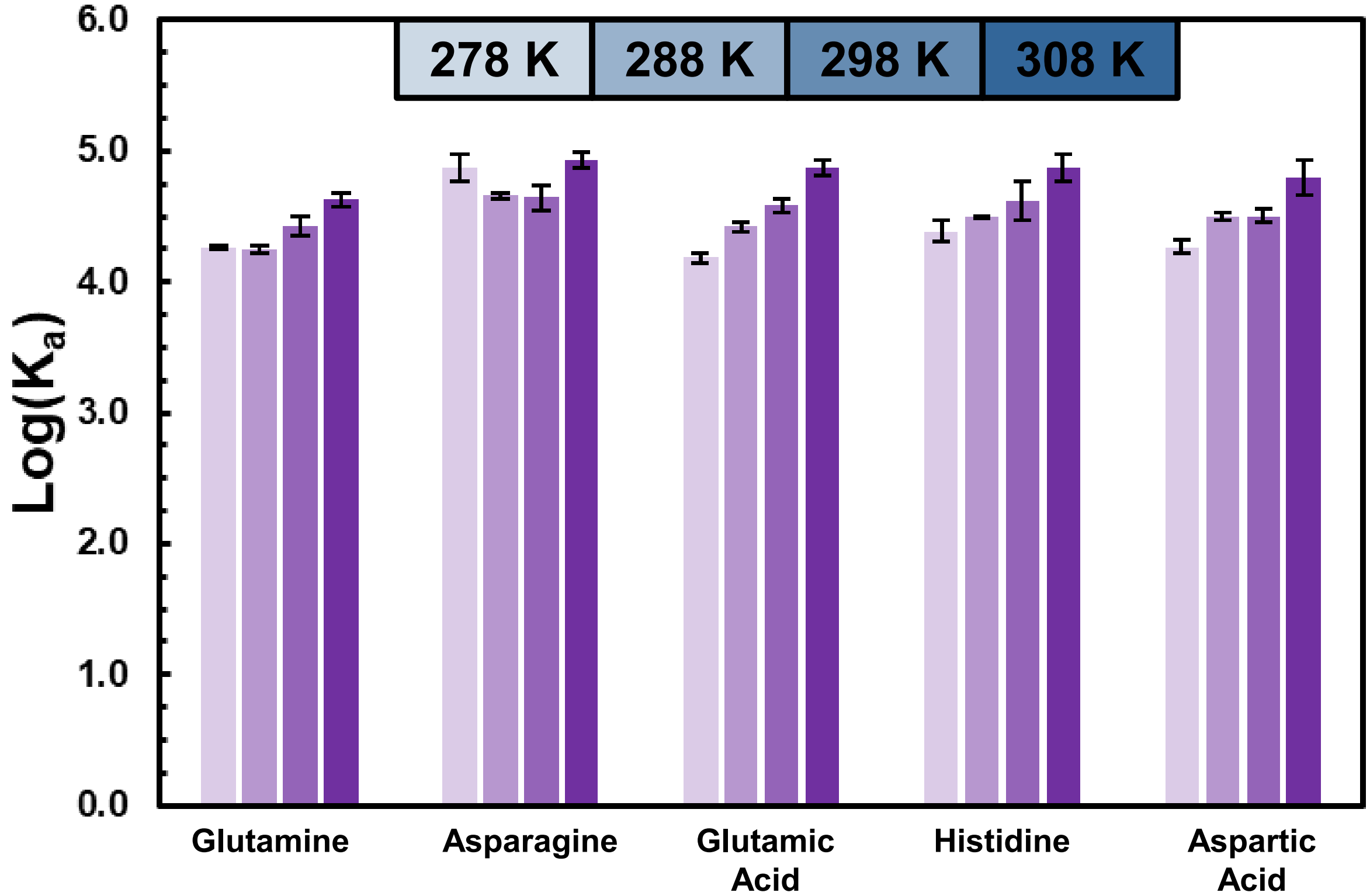

Figure S20. *Log (K_a) at 5, 15, 25, and 35 °C for Eu^{3+} binding by hydrophilic poly(amino acid acrylamides). Error bars are one standard deviation.*

References:

(1) Archer, W. R.; Gallagher, C. M. B.; Vaissier Welborn, V.; Schulz, M. D. Exploring the Role of Polymer Hydrophobicity in Polymer–Metal Binding Thermodynamics. *Phys. Chem. Chem. Phys.* **2022**, 10.1039.D1CP05263B. https://doi.org/10.1039/D1CP05263B.

(2) Turnbull, W. B.; Daranas, A. H. On the Value of *c* : Can Low Affinity Systems Be Studied by Isothermal Titration Calorimetry? *J. Am. Chem. Soc.* **2003**, *125* (48), 14859–14866. https://doi.org/10.1021/ja036166s.

(3) Mizoue, L. S.; Tellinghuisen, J. The Role of Backlash in the "First Injection Anomaly" in Isothermal Titration Calorimetry. *Anal. Biochem.* **2004**, *326* (1), 125–127. https://doi.org/10.1016/j.ab.2003.10.048.

(4) Wiseman, T.; Williston, S.; Brandts, J. F.; Lin, L.-N. Rapid Measurement of Binding Constants and Heats of Binding Using a New Titration Calorimeter. *Anal. Biochem.* **1989**, *179* (1), 131–137. https://doi.org/10.1016/0003-2697(89)90213-3.

(5) Wolfenden, R.; Andersson, L.; Cullis, P. M.; Southgate, C. C. B. Affinities of Amino Acid Side Chains for Solvent Water. *Biochemistry* **1981**, *20* (4), 849–855. https://doi.org/10.1021/bi00507a030.
